# Regional phenotypic surveillance of antimicrobial susceptibility in chicken-associated commensal *Staphylococcus*, *Enterococcus*, and *Escherichia coli* from Southern Transdanubia, Hungary

**DOI:** 10.3389/fvets.2026.1873052

**Published:** 2026-07-02

**Authors:** Ádám Kerek, Levente Hunor Husz, Edit Szarka, Gergely Álmos Tornyos, Franciska Barnácz, Bence Csirmaz, László Kovács, Ákos Jerzsele

**Affiliations:** 1 Department of Pharmacology and Toxicology, University of Veterinary Medicine, Budapest, Hungary; 2 National Laboratory of Infectious Animal Diseases, Antimicrobial Resistance, Veterinary Public Health and Food Chain Safety, University of Veterinary Medicine, Budapest, Hungary; 3 Department of Animal Hygiene, Herd Health and Mobile Clinic, University of Veterinary Medicine, Budapest, Hungary

**Keywords:** antimicrobial resistance, antimicrobial susceptibility, broiler chickens, commensal bacteria, *Enterococcus*, *Escherichia coli*, phenotypic surveillance, *Staphylococcus*

## Abstract

**Background:**

Antimicrobial resistance in commensal bacteria from poultry is an important indicator of selection pressure within food-animal production systems and may contribute to the broader One Health burden of antimicrobial resistance. Region-specific phenotypic surveillance is therefore needed to characterize antimicrobial susceptibility patterns in bacterial populations associated with intensive poultry production. The objective of this study was to characterize region-specific phenotypic antimicrobial susceptibility and co-resistance patterns in chicken-associated commensal *Staphylococcus*, *Enterococcus*, and *Escherichia coli* isolates from large-scale poultry flocks in Southern Transdanubia, Hungary.

**Methods:**

This study assessed the antimicrobial susceptibility profiles of chicken-associated commensal *Staphylococcus*, *Enterococcus*, and *Escherichia coli* isolates collected from large-scale flocks in Southern Transdanubia, Hungary. In total, 198 isolates, comprising *Staphylococcus* spp. (*n* = 40), *Enterococcus* spp. (*n* = 84), and *E. coli* (*n* = 74), were examined by broth microdilution to determine minimum inhibitory concentrations against a panel of antimicrobial agents relevant to veterinary and public health surveillance. Interpretive classifications were applied only where appropriate clinical breakpoints or epidemiological cut-off values were available; otherwise, MIC distributions were reported descriptively.

**Results:**

Multidrug-resistant phenotypes were frequent, occurring in 65.0% of *Staphylococcus*, 73.8% of *Enterococcus*, and 82.4% of *E. coli* isolates, with the highest proportions observed among *E. coli* and *Enterococcus*. Exploratory correlation, clustering, and network-based analyses indicated structured phenotypic co-resistance patterns, suggesting repeated co-occurrence of reduced susceptibility to selected antimicrobial classes within the tested isolate collection.

**Conclusion:**

These associations should not be interpreted as direct evidence of genetic linkage, horizontal gene transfer, or shared resistance determinants, but they may indicate possible co-selection requiring confirmation by future genomic studies. Overall, this study provides region-specific baseline data on antimicrobial susceptibility among chicken-associated commensal bacteria from Hungary and supports the need for harmonized phenotypic surveillance, prudent antimicrobial use, and genotype-informed follow-up investigations within a One Health framework.

## 1 Introduction

Antimicrobial resistance (AMR) remains one of the most pressing challenges at the interface of human, animal, and environmental health, driven in part by the overlapping use of antimicrobial agents across clinical, veterinary, and agricultural settings (1). Poultry meat is among the most widely consumed animal-derived protein sources worldwide. According to joint projections by the Organization for Economic Co-operation and Development (OECD) and the Food and Agriculture Organization (FAO), global poultry consumption is expected to increase by 17.8% by 2030, making it the fastest-growing meat category globally (2). This expansion is supported by advances in genetics, nutrition, production efficiency, and flock health management, but it also reinforces the need for robust surveillance of bacterial populations associated with intensive poultry systems (3).

Antimicrobial agents have played an important role in maintaining poultry health and controlling bacterial infections; however, their use can also contribute to the selection and persistence of resistant bacterial populations. In response to increasing concerns regarding AMR, particularly resistance involving antimicrobials of critical importance to human medicine, many countries have implemented restrictions or stewardship programs aimed at reducing unnecessary antimicrobial use in food-producing animals. At the same time, alternative strategies, including improved biosecurity, vaccination, probiotics, phytogenic compounds, organic acids, antimicrobial peptides, and other non-antibiotic interventions, have gained increasing attention as complementary tools to mitigate AMR in poultry production (4–13). Despite these efforts, multidrug-resistant bacteria continue to be reported in poultry-associated bacterial populations, highlighting the need for sustained phenotypic surveillance and region-specific risk assessment (14, 15).

The gastrointestinal tract of poultry is a complex microbial ecosystem that contributes to nutrient utilization, immune development, colonization resistance, and overall flock health (16). Recent studies and reviews have further emphasized that the composition and functional capacity of the chicken gut microbiome are shaped by age, diet, production system, environmental exposure, and antimicrobial use, all of which may influence the resistome and the phenotypic resistance patterns detectable in commensal bacteria (17, 18). Its microbiota includes a broad range of bacterial taxa, including *Lactobacillus*, *Clostridium*, *Ruminococcus*, *Enterococcus*, and *Escherichia*. Commensal bacteria within this ecosystem are particularly relevant for AMR monitoring because they may reflect antimicrobial selection pressure at the flock level and may serve as indicators of resistance dynamics within food-animal production systems. Mobile genetic elements, including plasmids and transposons, can facilitate the dissemination of antimicrobial resistance determinants among bacterial populations; however, phenotypic susceptibility data alone cannot confirm the presence, location, or transferability of such determinants. Therefore, MIC-based surveillance remains an essential first-line approach for identifying resistance patterns that warrant further molecular investigation (19).

*Enterococcus* species are of particular interest in veterinary AMR surveillance because of their intrinsic resistance traits, environmental persistence, and capacity to acquire additional antimicrobial resistance determinants (20). Members of this genus include clinically relevant organisms, such as *Enterococcus faecium* and *Enterococcus faecalis*, which are also represented among priority nosocomial pathogens in human medicine (21, 22). In poultry production, enterococci are commonly recovered from intestinal samples and can provide valuable information on resistance trends affecting Gram-positive commensal bacteria. The occurrence of multidrug-resistant *Enterococcus* spp. in poultry remains a concern despite increasingly restrictive antimicrobial use policies. According to the European Food Safety Authority (EFSA), *E. faecalis*, together with *E. coli*, is among the main AMR-associated bacterial indicators monitored in EU poultry populations, with particular attention to resistance affecting antimicrobial classes relevant to both veterinary and public health surveillance (23).

*Staphylococcus* species are Gram-positive bacteria that may occur as commensals but are also capable of causing opportunistic infections in birds and humans. In poultry, staphylococcal infections may manifest as salpingitis, gangrenous dermatitis, arthritis, or other localized and systemic disease conditions (24–26). Although the public health relevance of poultry-associated *Staphylococcus* spp. varies according to species, resistance phenotype, and exposure route, recent poultry-focused studies indicate that *Staphylococcus* spp., including *S. aureus*, may occur in chicken flocks, poultry meat, and processing environments and may carry phenotypic resistance profiles relevant to food-chain and occupational exposure (27, 28). Consequently, monitoring antimicrobial susceptibility in poultry-associated staphylococci can provide useful complementary information for assessing Gram-positive resistance patterns in intensive poultry production systems (29, 30).

*Escherichia coli* is a Gram-negative bacterium that is widely present as a commensal organism in the intestinal tract of chickens. However, pathogenic variants, collectively referred to as avian pathogenic *E. coli* (APEC), can cause colibacillosis and other systemic infections associated with substantial economic losses in poultry production (31–33). Colibacillosis remains one of the major bacterial disease challenges in commercial poultry systems worldwide (34). In addition to its clinical relevance, commensal *E. coli* is widely used as an indicator organism in AMR surveillance because it can reflect antimicrobial selection pressure in food-producing animals and may carry resistance phenotypes relevant to public health. The European Union AMR surveillance reports have repeatedly identified high levels of fluoroquinolone resistance and multidrug resistance among poultry-associated *E. coli*, supporting the continued need for standardized monitoring in this bacterial species (35, 36).

Monitoring antimicrobial susceptibility in commensal bacterial populations is essential for understanding the regional distribution of phenotypic resistance and for informing antimicrobial stewardship within a One Health framework. Resistance patterns may differ substantially between regions because of variation in antimicrobial use practices, production systems, biosecurity standards, farm management, and local bacterial ecology. Accordingly, this study aimed to characterize the phenotypic antimicrobial susceptibility profiles of chicken-associated commensal *Staphylococcus*, *Enterococcus*, and *E. coli* isolates collected from large-scale flocks in Southern Transdanubia, Hungary. By integrating MIC distributions with exploratory analyses of multidrug resistance and phenotypic co-resistance, the study provides region-specific baseline data to support veterinary AMR surveillance and to identify patterns that may warrant future genotype-informed investigation.

## 2 Materials and methods

### 2.1 Study design, sampling framework, and origin of isolates

This study was designed as a regional phenotypic antimicrobial susceptibility survey of commensal bacteria associated with large-scale chicken flocks in Southern Transdanubia, Hungary. The analysis included bacterial isolates obtained from three commercial chicken farms located within the Southern Transdanubian region. Farms were enrolled voluntarily and remained anonymized throughout the study. The selection of farms was intended to provide region-specific baseline information on antimicrobial susceptibility patterns in intensive chicken production; therefore, the results should be interpreted as regional surveillance data rather than as nationally representative estimates.

The participating farms were conventional large-scale commercial chicken farms with comparable intensive management systems. Farms were eligible for inclusion if they were located in Southern Transdanubia, operated under large-scale commercial production conditions, allowed routine veterinary sampling, and provided anonymized metadata on production type, age category, and flock-size category. No farm was selected on the basis of previous antimicrobial resistance results.

Samples were collected between 2022 and 2023 during routine veterinary sampling performed by veterinarians serving the participating farms, in collaboration with the Department of Animal Hygiene, Herd Health and Mobile Clinic, University of Veterinary Medicine Budapest. Sampling was conducted with the consent of farm operators and did not involve any experimental treatment, intervention, or research-induced manipulation of animals. Within each participating flock, clinically healthy birds showing no overt signs of infectious disease were selected randomly by the attending veterinarian from different areas of the poultry house to reduce pen-level or location-related sampling bias. Birds from flocks reported by the attending veterinarian to be under active antimicrobial treatment at the time of sampling were not included; however, detailed historical antimicrobial use data were not available and this is acknowledged as a limitation.

On each farm, 15 clinically healthy birds were sampled, and one cloacal and one tracheal swab were collected from each bird, resulting in 30 swab samples per farm and 90 swab samples in total. From each culture-positive sample, one purified isolate per target bacterial group was retained for further analysis. The final isolate collection comprised 40 *Staphylococcus* spp., 84 *Enterococcus* spp., and 74 *E. coli* isolates included in the phenotypic antimicrobial susceptibility analysis.

### 2.2 Bacterial isolation, preservation, and species identification

Bacterial isolation was performed at the Microbiology Laboratory of the Department of Pharmacology and Toxicology, University of Veterinary Medicine Budapest. Selective and differential culture media were used for the recovery of the target bacterial groups. Staphylococci were isolated on CHROMagar Staph aureus medium (Chebio Fejlesztő Kft., Budapest, Hungary), *Escherichia coli* isolates were recovered on ChromoBio Coliform agar (Biolab Zrt., Budapest, Hungary), and enterococci were isolated using m-Enterococcus modified agar (Merck KGaA, Darmstadt, Germany).

Presumptive colonies were subcultured on tryptic soy agar (Biolab Zrt., Budapest, Hungary) to obtain pure cultures. Purified isolates were stored in Microbank cryovials (Pro-Lab Diagnostics, Richmond Hill, Canada) at −80 °C until further analysis.

Species identification was performed by matrix-assisted laser desorption/ionization time-of-flight mass spectrometry (MALDI-TOF MS) using a Bruker MALDI Biotyper system and Biotyper software version 12.0 (Bruker Daltonics GmbH, Bremen, Germany, 2024), according to the manufacturer’s instructions (37). Each isolate was assigned a unique laboratory identification code before antimicrobial susceptibility testing. The final dataset comprised 198 isolates, including *Staphylococcus* spp. (*n* = 40), *Enterococcus* spp. (*n* = 84), and *E. coli* (*n* = 74). Species-level identification was accepted according to the manufacturer’s recommended MALDI-TOF MS score thresholds. Species-level identification was accepted according to the manufacturer’s recommended MALDI-TOF MS score thresholds. All isolates included in the final dataset were identified to species level before antimicrobial susceptibility testing.

### 2.3 Antimicrobial agents and preparation of stock solutions

Antimicrobial susceptibility testing was performed against a panel of antimicrobial agents selected to represent compounds of veterinary relevance and agents of public health importance for phenotypic surveillance. The panel included β-lactams (amoxicillin, amoxicillin–clavulanic acid, ceftriaxone, and imipenem), tetracyclines (doxycycline), aminoglycosides (neomycin and spectinomycin), fluoroquinolones (enrofloxacin), phenicols (florfenicol), macrolides/lincosamides or pleuromutilins where applicable (tylosin, lincomycin, and tiamulin), potentiated sulfonamides (trimethoprim–sulfamethoxazole, 1:19), glycopeptides (vancomycin), and polymyxins (colistin), depending on the bacterial group tested. The antimicrobial concentration ranges used for broth microdilution are shown in Supplementary Tables 1–S3 for each bacterial group, where the tested two-fold dilution series are presented together with MIC_50_ and MIC_90_ values.

Antimicrobial powders were obtained from Merck KGaA (Darmstadt, Germany). Stock solutions were prepared according to the recommendations of the Clinical and Laboratory Standards Institute (CLSI) for broth microdilution testing (38). Amoxicillin, amoxicillin–clavulanic acid at a 2:1 ratio, and imipenem were dissolved in phosphate buffer. Ceftriaxone, doxycycline, spectinomycin, neomycin, colistin, tiamulin, tylosin, lincomycin, and vancomycin were dissolved in distilled water. For trimethoprim-sulfamethoxazole, tested at a 1:19 ratio, sulfamethoxazole was dissolved in hot water with a small amount of sodium hydroxide, whereas trimethoprim was dissolved in hydrochloric acid diluted in distilled water. Enrofloxacin was prepared in distilled water with a small amount of sodium hydroxide, and florfenicol was dissolved using a small volume of 95% ethanol followed by dilution in distilled water.

Stock solutions were adjusted according to manufacturer-stated potency and stored under conditions appropriate for each compound until use. Two-fold serial dilutions were prepared to generate the concentration ranges required for MIC determination.

### 2.4 Broth microdilution MIC testing

Minimum inhibitory concentrations (MICs) were determined using the broth microdilution method in 96-well microtiter plates, following CLSI principles for aerobic bacteria (38). Testing was performed in cation-adjusted Mueller–Hinton broth unless otherwise required for a specific organism–antimicrobial combination.

Frozen isolates were revived from Microbank cryovials by inoculation into cation adjusted Mueller–Hinton broth and incubation at 37 °C for 18–24 h. Fresh cultures were adjusted to the turbidity of a 0.5 McFarland standard and subsequently diluted to obtain the final inoculum concentration required for broth microdilution testing. Antimicrobial dilutions were prepared in 96-well plates using two-fold serial dilution across the test range. Positive growth controls containing inoculated broth without antimicrobial agent and negative sterility controls containing uninoculated broth were included on each plate.

Plates were incubated at 37 °C for 18–24 h under aerobic conditions. MICs were read as the lowest antimicrobial concentration showing no visible bacterial growth. MIC determination was supported by the SWIN automated MIC reading system and the VIZION digital viewing system (CheBio Fejlesztő Kft., Budapest, Hungary). Quality control was performed using reference strains appropriate for the bacterial groups and antimicrobial agents tested, including *E. coli* ATCC 25922, *Enterococcus faecalis* ATCC 29212, and *Staphylococcus aureus* ATCC 29213. Quality-control MIC values were evaluated against accepted reference ranges where available. MIC testing was performed once for each isolate under standardized conditions. Quality-control strains were included on each testing day, and isolates with unclear growth endpoints or unexpected results were retested.

### 2.5 Interpretation of MIC results

MIC distributions were summarized for each bacterial group and antimicrobial agent. Interpretive categories were assigned only when appropriate clinical breakpoints or epidemiological cut-off values were available from recognized standards, including CLSI M07 and the EUCAST breakpoint tables and epidemiological cut-off values available in 2023 (38, 39). Where species-specific or host-relevant clinical breakpoints were available, isolates were categorized according to the corresponding interpretive criteria. Where only epidemiological cut-off values were available, isolates were classified as wild-type or non-wild-type rather than clinically susceptible or resistant.

For antimicrobial–bacterial group combinations for which no appropriate clinical breakpoint or epidemiological cut-off value was available, MIC values were reported descriptively and were not converted into clinical resistance categories. In such cases, MIC values were described as MIC distributions, and the term “reduced susceptibility” was used only in a descriptive phenotypic sense when supported by the observed MIC pattern. The term “elevated MIC” was reserved for descriptive discussion of MIC distributions and was not used as a clinical interpretive category. This approach was adopted to avoid overinterpretation of MIC results for combinations lacking validated interpretive criteria in poultry-associated commensal isolates.

Multidrug-resistant phenotypes were defined according to the framework proposed by Magiorakos et al. (40) as reduced susceptibility or resistance to at least one antimicrobial agent in three or more antimicrobial classes among the tested compounds, where valid interpretive criteria allowed classification. MDR estimates were therefore interpreted within the limits of the antimicrobial panel and the available interpretive criteria. Extensively drug-resistant and pan-drug-resistant classifications were not used as primary outcome measures, because the tested panel was not designed to support standardized XDR or PDR categorization across all bacterial groups.

### 2.6 Descriptive and exploratory statistical analyses

All statistical analyses were performed using R software version 4.5.2. MIC distributions were summarized by bacterial group and antimicrobial agent. For each antimicrobial compound, MIC ranges, MIC_50_, and MIC_90_ values were calculated where appropriate. The proportions of isolates assigned to resistant or non-wild-type categories were calculated only for antimicrobial–bacterial group combinations with valid interpretive criteria.

Phenotypic co-resistance patterns were explored using Spearman’s rank correlation analysis. Correlation analyses were performed on binary phenotype matrices generated from interpretable antimicrobial susceptibility categories. Antimicrobial agents with no phenotypic variation within a given bacterial group were excluded from correlation analysis to avoid unstable or non-informative estimates. Correlation matrices were visualized as heatmaps to identify patterns of co-occurring reduced susceptibility. Spearman’s rank correlation analysis was used as an exploratory visualization approach to identify pairwise co-occurrence patterns among reduced-susceptibility phenotypes. Because the analysis involved multiple antimicrobial pairs within relatively small and uneven phenotype matrices, formal inferential testing of individual correlations was not used as a primary analytical objective. Accordingly, correlation coefficients were interpreted descriptively to support visualization of phenotypic co-resistance patterns rather than as confirmatory statistical evidence.

Principal component analysis was used as an exploratory visualization tool to assess the multivariate structure of phenotypic susceptibility profiles within each bacterial group. Clustering analyses were used only descriptively to visualize potential grouping of isolates based on phenotypic patterns and were not interpreted as evidence of distinct genetic lineages or transmission clusters.

For visualization, isolates were grouped using *k*-means clustering based on scaled binary susceptibility phenotype matrices, with the number of clusters set to three to provide a consistent descriptive visualization across bacterial groups. Clustering was used only as an exploratory graphical aid and was not interpreted as evidence of genetic relatedness or epidemiological clustering.

Network-based co-resistance visualization was performed to illustrate the frequency of concurrent reduced susceptibility to pairs of antimicrobial agents. In these graphs, nodes represented antimicrobial agents, and edges represented co-occurrence of reduced susceptibility within the same isolates. Node size was proportional to the number of isolates with reduced susceptibility to the corresponding antimicrobial agent, whereas edge thickness reflected the frequency of pairwise co-occurrence. These analyses were considered exploratory and hypothesis-generating.

No genetic linkage, horizontal gene transfer, plasmid carriage, or resistance-gene co-localization was inferred from phenotypic data alone. Associations observed in correlation, clustering, or network analyses were interpreted as phenotypic co-resistance patterns that may warrant future genomic investigation.

### 2.7 Ethical considerations

The study used bacterial isolates obtained during routine veterinary sampling from commercial chicken farms. No animals were sampled exclusively for the purposes of this study, and no experimental procedures, treatments, or interventions were performed. Sampling was carried out by veterinarians as part of routine flock-health monitoring with the consent of farm operators. Farm identities were anonymized before analysis, and only region-level information was retained for reporting. On this basis, formal animal experimentation approval was not required. The study was conducted in accordance with professional veterinary standards and institutional requirements for the use of bacterial isolates derived from routine diagnostic or surveillance activities.

## 3 Results

### 3.1 Isolate collection and overall dataset

A total of 198 chicken-associated commensal bacterial isolates from large-scale flocks in Southern Transdanubia, Hungary, were included in the phenotypic antimicrobial susceptibility analysis. The isolate collection comprised *Staphylococcus* spp. (*n* = 40), *Enterococcus* spp. (*n* = 84), and *Escherichia coli* (*n* = 74). All isolates were identified by MALDI-TOF MS before antimicrobial susceptibility testing. At species level, the *Staphylococcus* collection consisted mainly of *Staphylococcus aureus* (27/40, 67.5%), *Staphylococcus gallinarum* (8/40, 20.0%), and *Staphylococcus delphini* (5/40, 12.5%). The *Enterococcus* collection included *Enterococcus faecalis* (38/84, 45.2%), *Enterococcus faecium* (20/84, 23.8%), *Enterococcus gallinarum* (11/84, 13.1%), *Enterococcus durans* (9/84, 10.7%), *Enterococcus hirae* (4/84, 4.8%) and *Enterococcus mundtii* (2/84, 2.4%). All *E. coli* isolates were identified as *Escherichia coli* (*n* = 74). The complete species-level identification results are provided in Supplementary Tables 1–S3.

**Figure 1:**
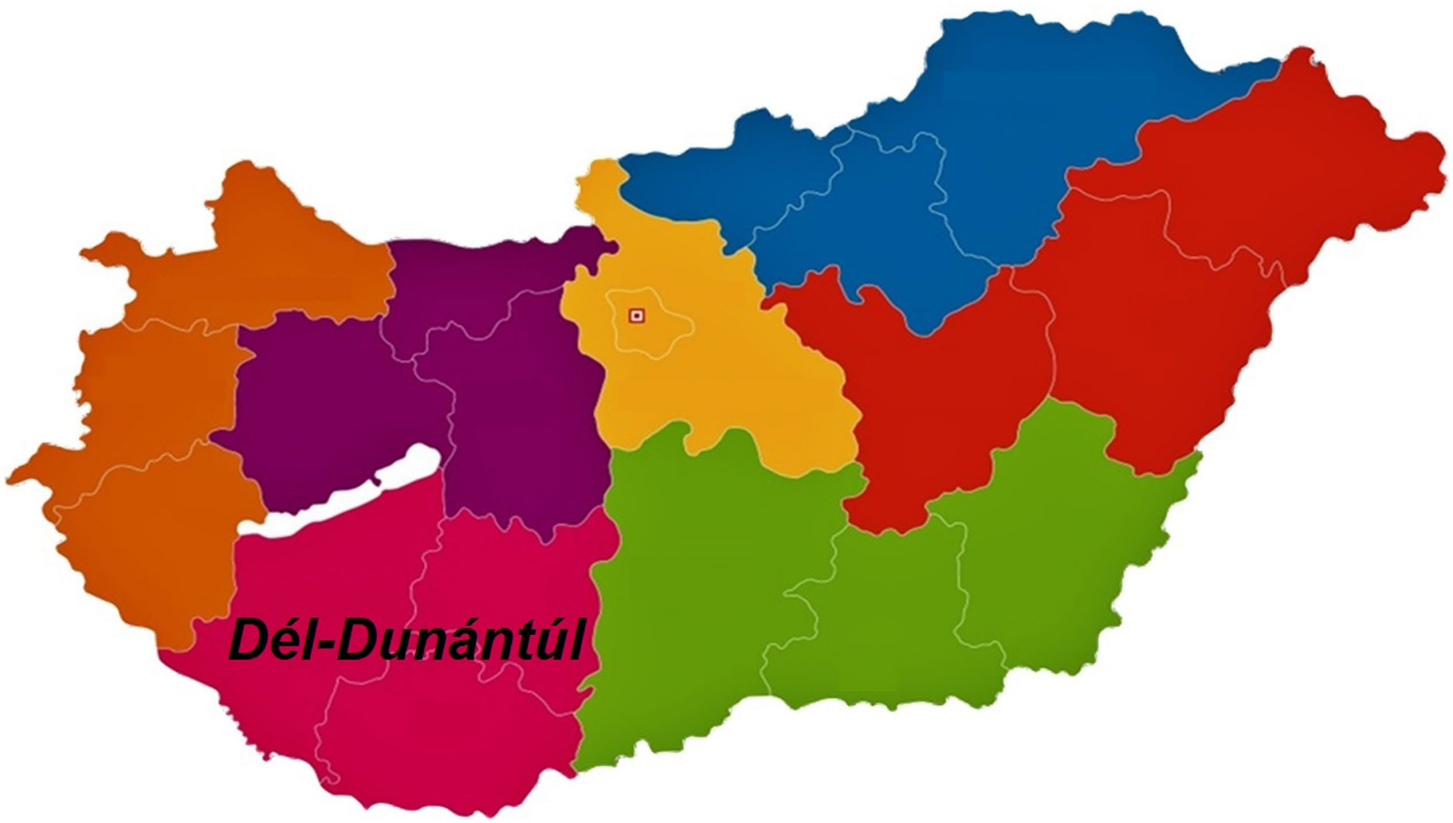
Map of Hungary showing its administrative regions, with the Dél-Dunántúl (Southern Transdanubia) region highlighted in pink. This study focused on large-scale poultry farms located within this region.

The geographical focus of the study was the Southern Transdanubian region of Hungary, as shown in Figure 1. Antimicrobial susceptibility testing generated genus-specific MIC distributions for the tested antimicrobial agents. Because validated interpretive criteria were not available for every organism–antimicrobial combination, MIC values were interpreted using clinical breakpoints or epidemiological cut-off values only where appropriate. For combinations lacking suitable interpretive criteria, results are presented descriptively as MIC distributions.

Farm-level and anatomical-site comparisons were not performed because the study was designed as a regional phenotypic surveillance survey rather than a stratified farm-level prevalence study, and isolate numbers per farm and sampling site were not balanced for inferential comparison.

### 3.2 Phenotypic antimicrobial susceptibility profiles of *Staphylococcus* isolates

The MIC distributions of the *Staphylococcus* isolates are summarized in Supplementary Table 1. The tested isolates showed heterogeneous susceptibility profiles across the antimicrobial panel, with marked differences between compounds. High proportions of reduced susceptibility were observed for several agents used or monitored in poultry-associated bacterial populations, whereas low MIC values were recorded for imipenem and vancomycin in most isolates. The highest MIC_90_ values were observed for doxycycline (128 μg/mL), tylosin (1,024 μg/mL), potentiated sulfonamides (1,024 μg/mL), and tiamulin (64 μg/mL), whereas imipenem and vancomycin showed low MIC_90_ values of 1 and 2 μg/mL, respectively.

Among β-lactams, MICs for amoxicillin were frequently above the applied interpretive threshold, whereas MICs for amoxicillin–clavulanic acid were generally lower. This pattern is compatible with phenotypic β-lactamase-mediated reduced susceptibility in part of the isolate collection, but it was interpreted only as a phenotypic observation because no molecular confirmation was performed. Doxycycline MICs were also frequently elevated, and enrofloxacin showed a broad MIC distribution, indicating variable fluoroquinolone susceptibility among the *Staphylococcus* isolates. Tiamulin and tylosin likewise showed heterogeneous MIC patterns, suggesting considerable isolate-level variation in phenotypic susceptibility.

**Figure 2:**
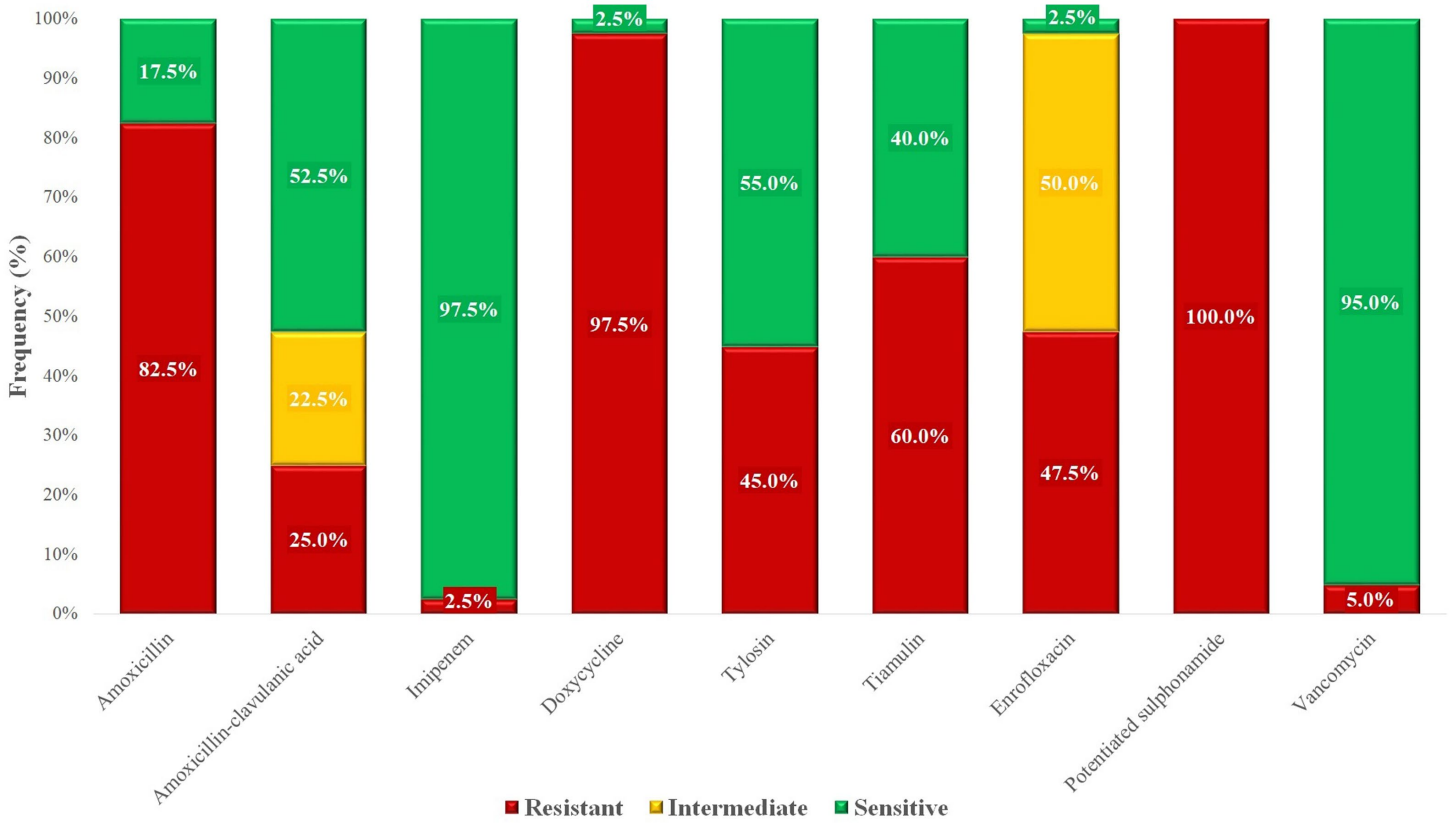
Phenotypic antimicrobial susceptibility profile of *Staphylococcus* isolates (*n* = 40) from broiler chickens in Southern Transdanubia, Hungary.

The overall antimicrobial susceptibility profile of the *Staphylococcus* isolates is shown in Figure 2. The highest proportions of reduced susceptibility were observed for potentiated sulfonamides (40/40, 100.0%), amoxicillin (33/40, 82.5%), doxycycline (39/40, 97.5%), tiamulin (24/40, 60.0%), and enrofloxacin (19/40, 47.5%). In contrast, most isolates displayed low MICs to imipenem and vancomycin, with reduced-susceptibility phenotypes detected in 1/40 (2.5%) and 2/40 (5.0%) isolates, respectively, according to the applied interpretive criteria. These results indicate that the *Staphylococcus* population recovered from chicken flocks in Southern Transdanubia was phenotypically diverse, with several isolates showing reduced susceptibility to multiple antimicrobial classes.

**Figure 3:**
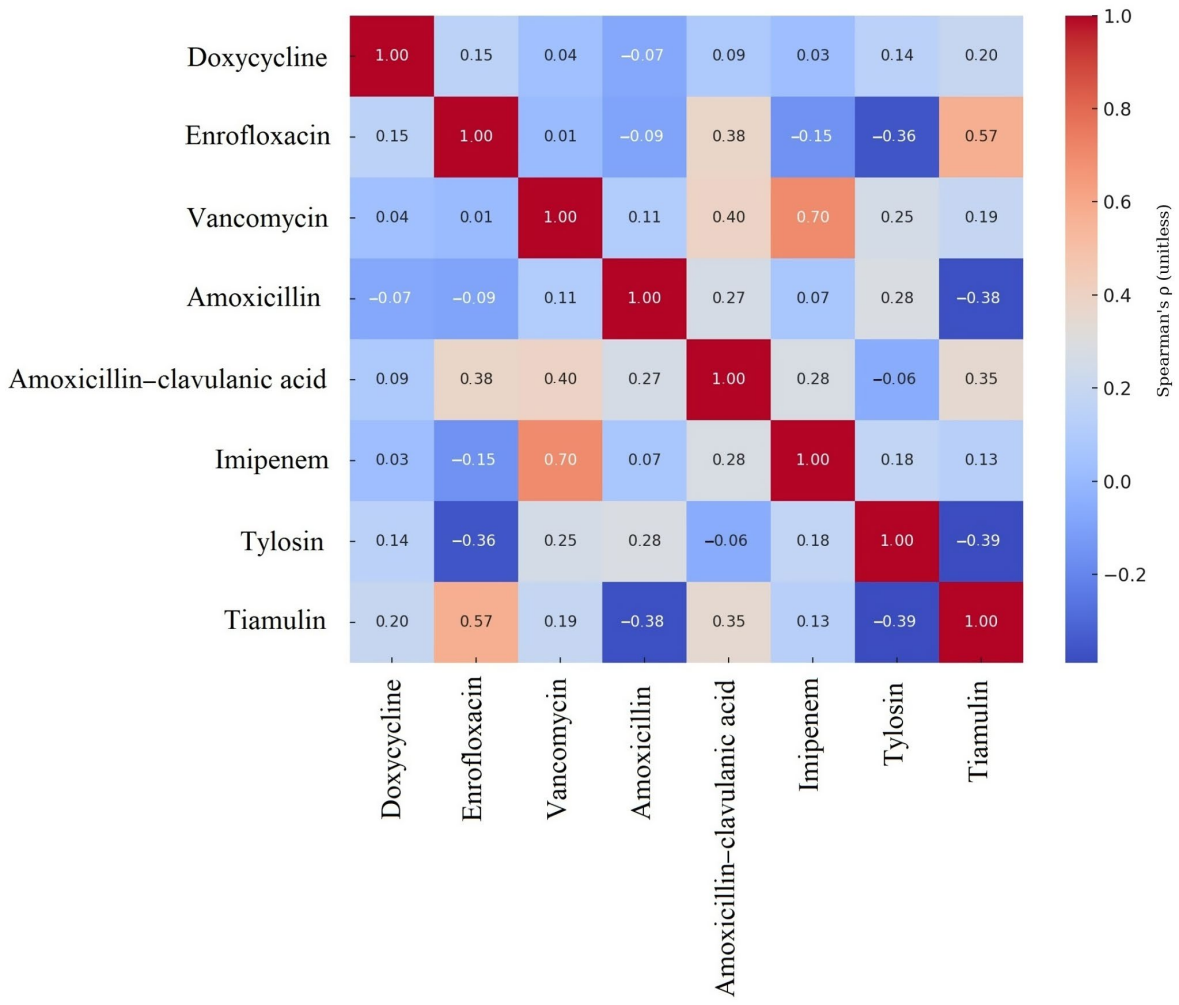
Spearman correlation heatmap of *Staphylococcus* isolates (*n* = 40) isolated from chickens in the Southern Transdanubia region. Positive values indicate co-occurrence of reduced susceptibility phenotypes, values near zero suggest weak or no association, and negative values indicate inverse phenotypic associations. The heatmap was interpreted descriptively and was used to visualize exploratory co-occurrence patterns rather than to provide confirmatory statistical evidence.

Exploratory Spearman correlation analysis was performed to evaluate co-occurrence patterns of reduced susceptibility among antimicrobial agents. Antimicrobial agents with no variation in phenotype were excluded from this analysis. In the *Staphylococcus* dataset, the strongest positive pairwise correlation coefficient was observed between vancomycin and imipenem reduced-susceptibility phenotypes (*r* = 0.70; Figure 3). This association was unexpected from a biological and veterinary-use perspective, because neither imipenem nor vancomycin is used in poultry production. Therefore, it was interpreted as a descriptive phenotypic co-occurrence pattern within this isolate collection rather than as evidence of shared selection pressure, causal association, or genetic linkage.

**Figure 4:**
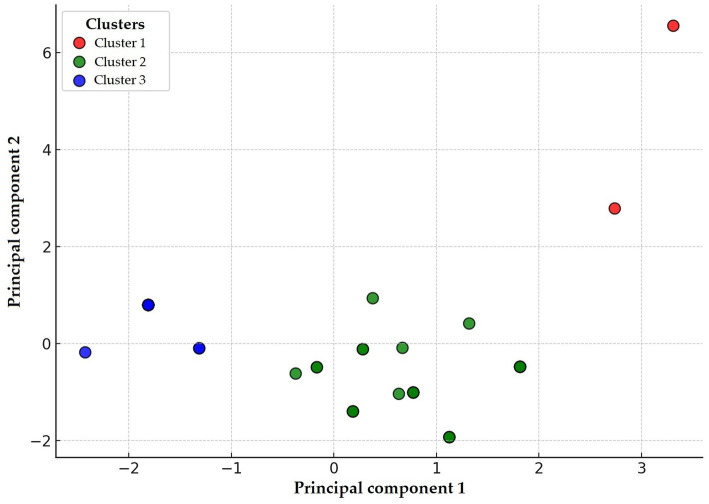
Principal component analysis (PCA) and clustering of *Staphylococcus* isolates (*n* = 40) from broiler chickens in the Southern Transdanubia region. The red, green, and blue represent clusters 1, 2, and 3, respectively.

Principal component analysis provided an exploratory visualization of multivariate susceptibility profiles among *Staphylococcus* isolates (Figure 4). The PCA plot suggested that isolates did not form a single homogeneous phenotypic population; instead, several isolates separated according to differences in their antimicrobial susceptibility profiles. These patterns support the presence of phenotypic heterogeneity within the *Staphylococcus* collection but should not be interpreted as evidence of clonal relatedness or distinct transmission lineages.

**Figure 5:**
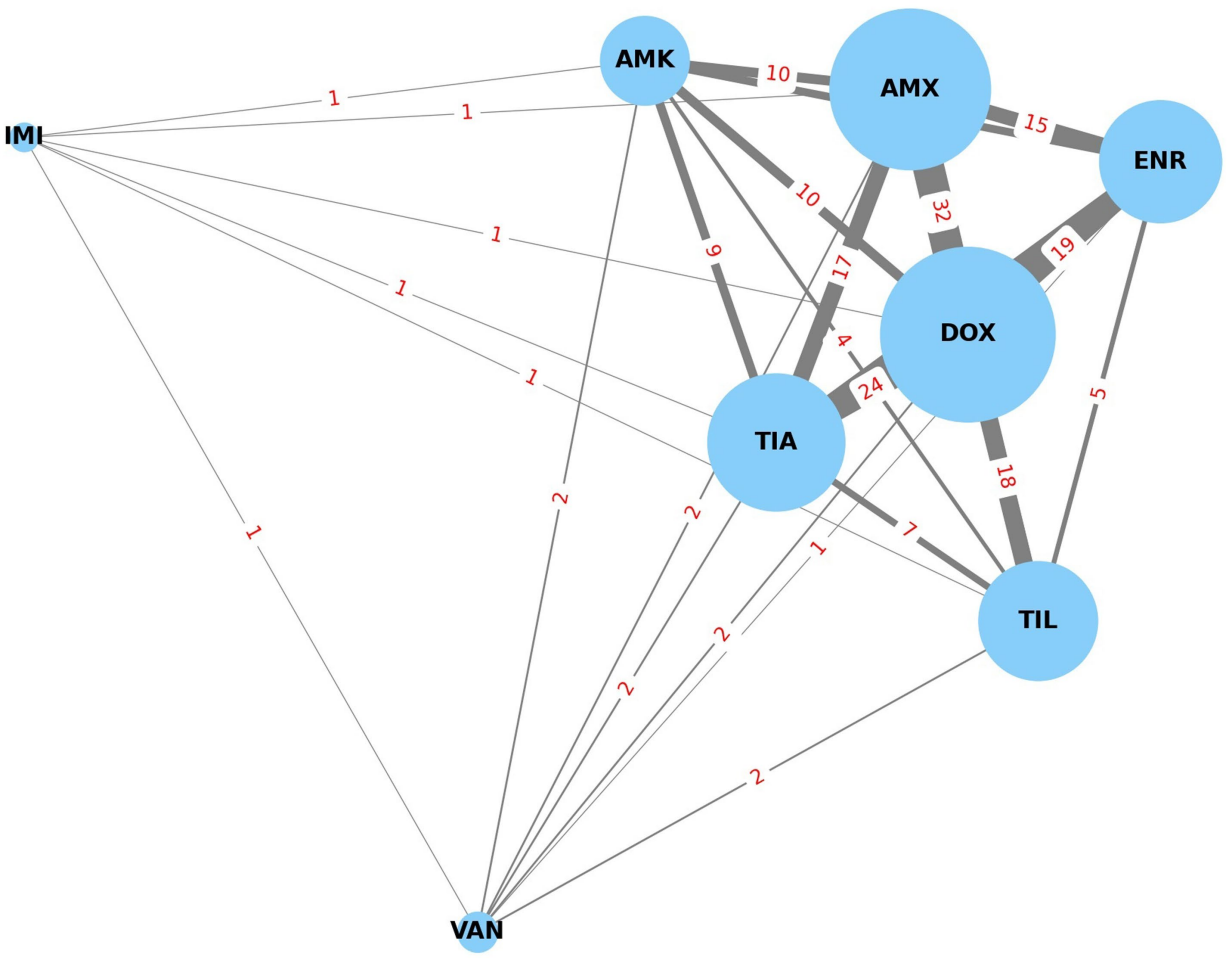
Phenotypic co-resistance network of *Staphylococcus* isolates (*n* = 40) from broiler chickens in the Southern Transdanubia region. AMX, amoxicillin; AMK, amoxicillin-clavulanic acid; DOX, doxycycline; TIL, tylosin; TIA, tiamulin; ENR, enrofloxacin; PSA, potentiated sulfonamide (trimethoprim-sulfamethoxazole, 1:19).

Network-based visualization further illustrated pairwise phenotypic co-resistance patterns among the tested antimicrobial agents (Figure 5). The network should be interpreted as a descriptive summary of co-occurrence patterns within the isolate collection rather than as evidence of shared genetic mechanisms.

To further explore whether selected antimicrobial phenotypes could separate MDR from non-MDR isolates, a decision tree model was generated as a supplementary, hypothesis-generating analysis. This model suggested that reduced susceptibility to selected agents contributed to the phenotypic separation of MDR isolates; however, the model was not used for clinical prediction and was not interpreted as evidence of causal resistance mechanisms (Supplementary Figure S1). A supplementary Monte Carlo analysis was also performed to contextualize the observed MDR frequency under a randomized phenotypic distribution, and the results are presented as an exploratory robustness check rather than as formal evidence of selection pressure (Supplementary Figure S2).

### 3.3 Phenotypic antimicrobial susceptibility profiles of Enterococcus isolates

The MIC distributions of the *Enterococcus* isolates are presented in Supplementary Table S2. The *Enterococcus* collection displayed broad MIC distributions for several antimicrobial agents, with particularly variable results for amoxicillin, doxycycline, enrofloxacin, florfenicol, neomycin, potentiated sulfonamides, tylosin, and vancomycin. MIC_90_ values were particularly high for amoxicillin (512 μg/mL), neomycin (1,024 μg/mL), potentiated sulfonamides (1,024 μg/mL), tylosin (1,024 μg/mL), and vancomycin (1,024 μg/mL), whereas florfenicol had a lower MIC_90_ of 32 μg/mL.

For amoxicillin, most isolates remained within lower MIC ranges, although a subset exhibited elevated MICs. Amoxicillin–clavulanic acid MICs were generally distributed toward lower or intermediate concentrations. Doxycycline and enrofloxacin showed wide MIC distributions, indicating variability in phenotypic susceptibility among isolates. Florfenicol, tylosin, and potentiated sulfonamides also showed substantial variability, with several isolates displaying elevated MIC values.

Vancomycin MICs were distributed across a broad range, and a subset of isolates showed elevated MICs according to the applied interpretive criteria. Because this study did not include molecular testing for vancomycin-resistance determinants, these findings should be interpreted strictly as phenotypic observations. Molecular characterization would be required to determine whether elevated vancomycin MICs are associated with known acquired resistance mechanisms.

**Figure 6:**
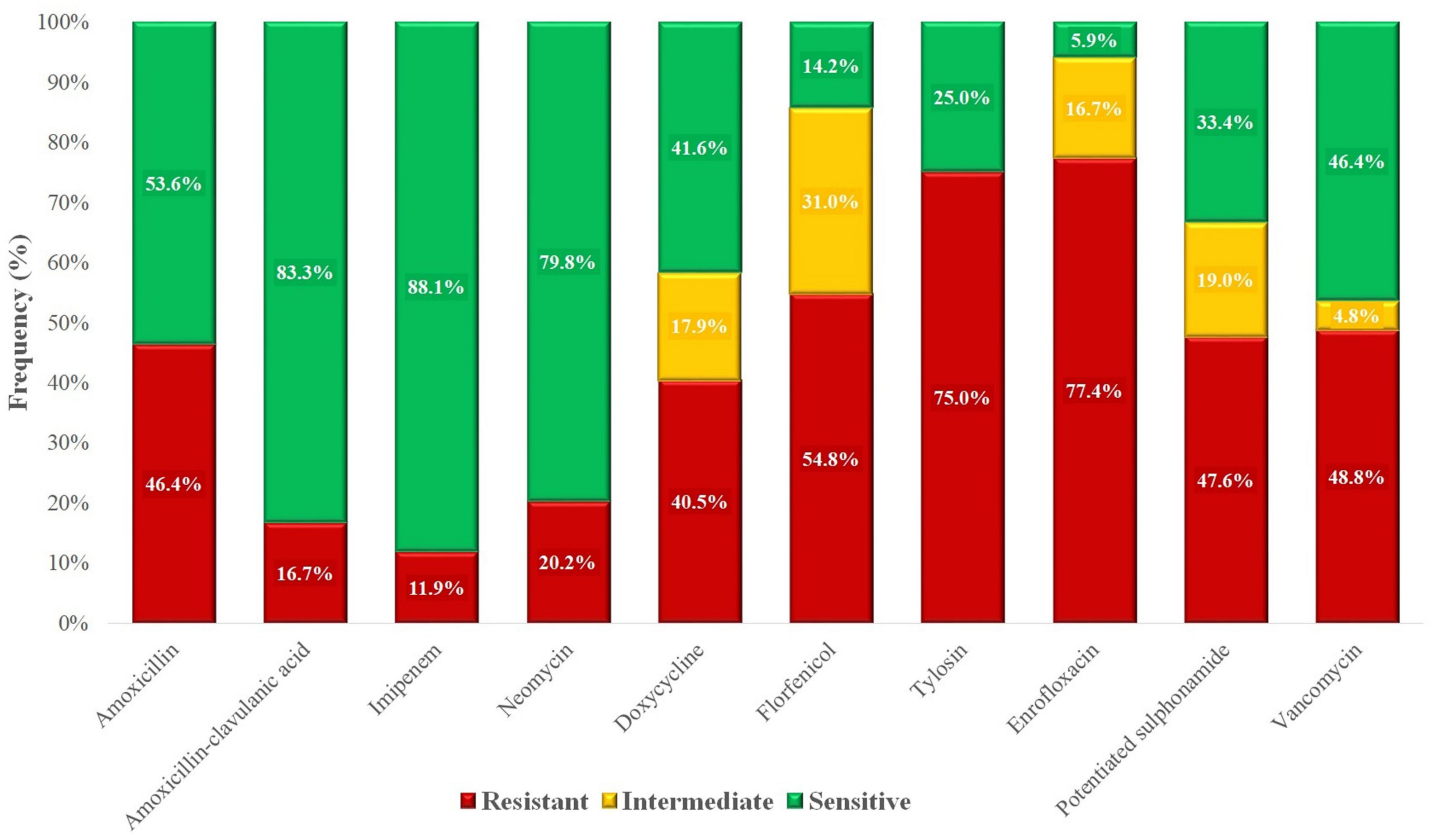
Phenotypic antimicrobial susceptibility profile of *Enterococcus* isolates (*n* = 84) from broiler chickens in Southern Transdanubia, Hungary.

The genus-level antimicrobial susceptibility profile of *Enterococcus* isolates is shown in Figure 6. The most frequent reduced-susceptibility phenotypes in the *Enterococcus* collection involved tylosin (63/84, 75.0%), enrofloxacin (65/84, 77.4%), potentiated sulfonamides (40/84, 47.6%), vancomycin (41/84, 48.8%), and neomycin (17/84, 20.2%), according to the applied interpretive criteria. However, the magnitude of reduced susceptibility differed by antimicrobial agent, underscoring the importance of compound-specific interpretation rather than broad generalization across antimicrobial classes.

**Figure 7:**
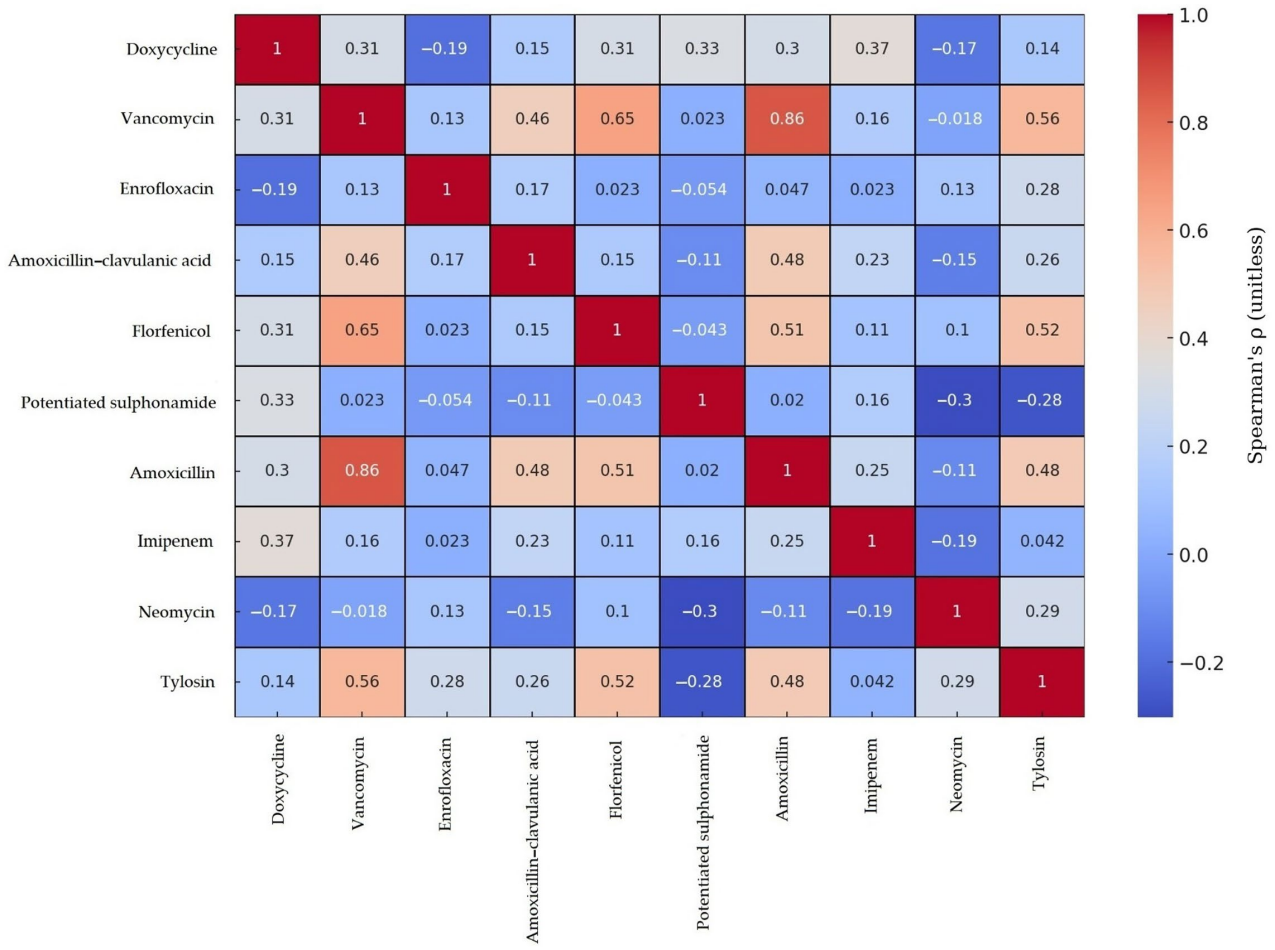
Spearman correlation heatmap of *Enterococcus* isolates (*n* = 84) isolated from broiler chickens in the Southern Transdanubia region. Positive values indicate co-occurrence of reduced susceptibility phenotypes, values near zero suggest weak or no association, and negative values indicate inverse phenotypic associations. The heatmap was interpreted descriptively and was used to visualize exploratory co-occurrence patterns rather than to provide confirmatory statistical evidence.

Exploratory correlation analysis identified positive phenotypic associations between selected antimicrobial agents (Figure 7). In particular, co-occurrence of reduced susceptibility was observed among several compounds with broad MIC distributions. These associations may reflect shared selection pressures or co-occurring phenotypes within the tested isolate collection, but they do not provide direct evidence of genetic linkage, mobile genetic elements, or horizontal gene transfer.

**Figure 8:**
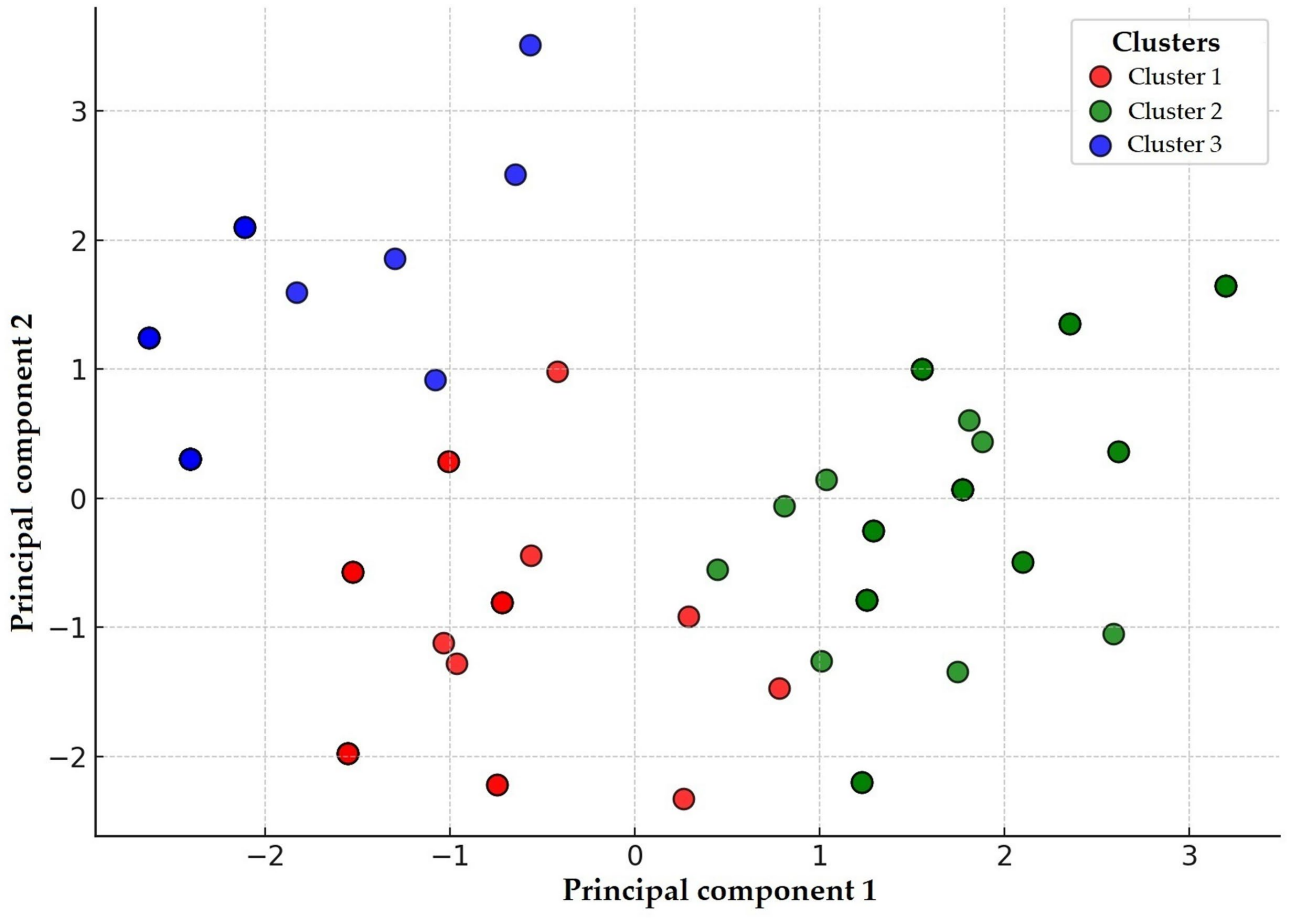
Principal component analysis (PCA) and clustering of *Enterococcus* isolates (*n* = 84) from broiler chickens in the Southern Transdanubia region. Red, green, and blue represent clusters 1, 2, and 3, respectively.

Principal component analysis of *Enterococcus* susceptibility profiles indicated phenotypic variation among isolates (Figure 8). The distribution of isolates in the PCA space suggested that reduced susceptibility patterns were not evenly distributed across the collection. Nevertheless, the PCA was used only as an exploratory tool to visualize phenotypic structure and not to infer genetic relationships.

**Figure 9:**
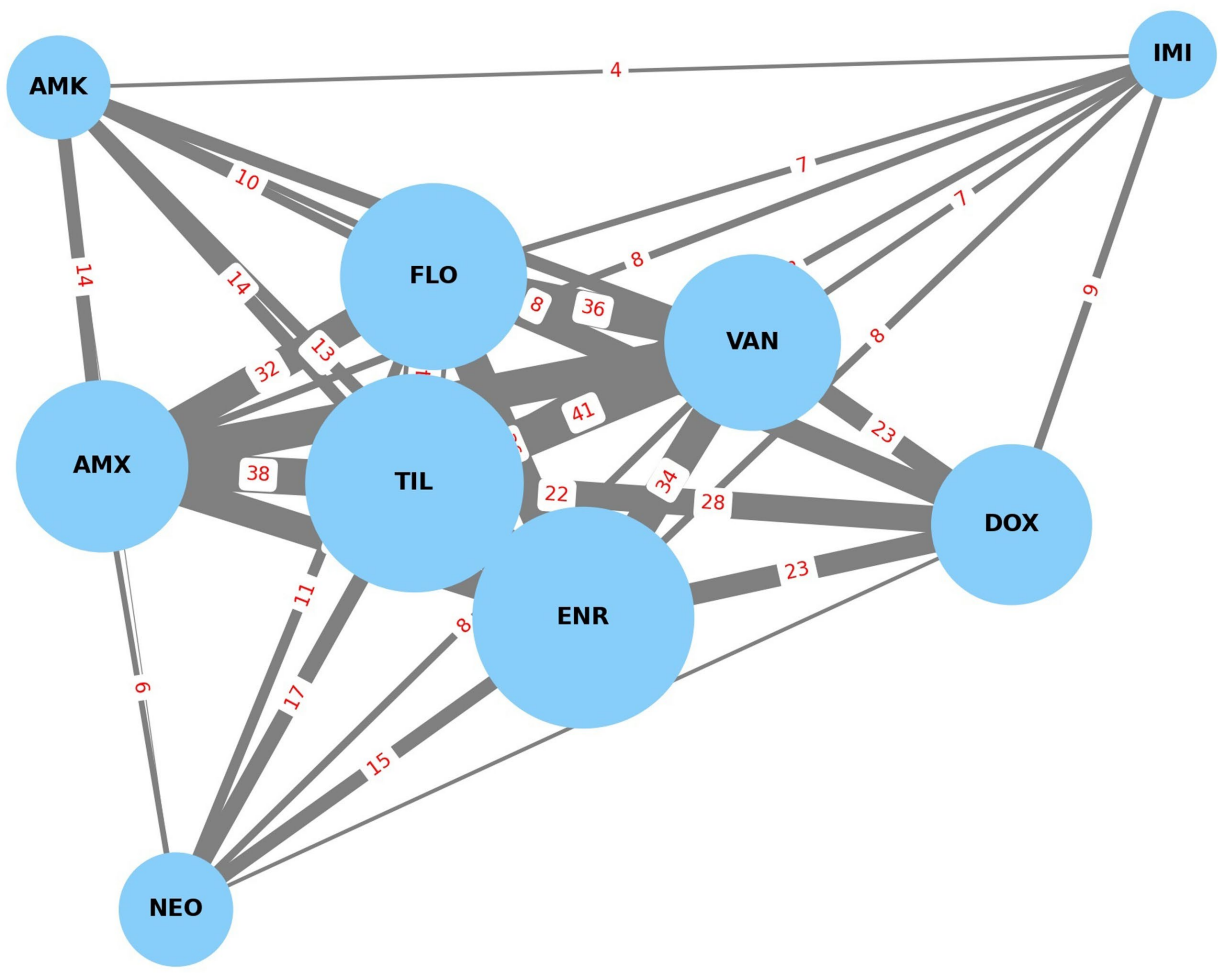
Phenotypic co-resistance network of *Enterococcus* isolates (*n* = 84) from broiler chickens in the Southern Transdanubia region. AMX, amoxicillin; AMK, amoxicillin-clavulanic acid; DOX, doxycycline; TIL, tylosin; TIA, tiamulin; ENR, enrofloxacin; PSA, potentiated sulfonamide (trimethoprim-sulfamethoxazole, 1:19).

Network-based visualization provided an additional descriptive representation of pairwise phenotypic co-resistance patterns among *Enterococcus* isolates (Figure 9). These patterns were considered exploratory and were not used to infer genetic linkage, mobile genetic elements, or horizontal gene transfer.

Supplementary exploratory analyses were performed to assess whether phenotypic reduced-susceptibility patterns could distinguish MDR from non-MDR *Enterococcus* isolates. The decision tree analysis identified selected antimicrobial phenotypes that contributed to MDR classification within this dataset, but the analysis was considered descriptive and was not used to infer causal relationships or genetic linkage (Supplementary Figure S3). A Monte Carlo simulation was additionally used to compare the observed MDR burden with randomized phenotypic distributions, providing supportive exploratory context for the non-uniform distribution of MDR phenotypes (Supplementary Figure S4).

### 3.4 Phenotypic antimicrobial susceptibility profiles of *Escherichia coli* isolates

The MIC distributions of the *E. coli* isolates are summarized in Supplementary Table S3. The *E. coli* collection showed high levels of reduced susceptibility to several antimicrobial agents relevant to poultry-associated Gram-negative bacteria. Broad MIC distributions were observed for amoxicillin, ceftriaxone, doxycycline, enrofloxacin, florfenicol, neomycin, potentiated sulfonamides, and spectinomycin. The highest MIC_90_ values in the *E. coli* collection were observed for amoxicillin (1,024 μg/mL), ceftriaxone (256 μg/mL), neomycin (256 μg/mL), potentiated sulfonamides (1,024 μg/mL), and spectinomycin (256 μg/mL), whereas colistin showed a low MIC_90_ of 0.5 μg/mL.

Amoxicillin MICs were frequently elevated, whereas amoxicillin–clavulanic acid MICs were generally lower. This phenotypic pattern may be compatible with β-lactamase-mediated reduced susceptibility in part of the *E. coli* population, although no molecular confirmation was performed. Ceftriaxone MICs were variable, with a subset of isolates showing elevated values. Because third-generation cephalosporin susceptibility is of particular public health relevance, these findings highlight the need for future genotype-informed analysis of β-lactam resistance mechanisms.

Enrofloxacin and florfenicol showed broad MIC distributions, and elevated MICs were common within the *E. coli* collection. Neomycin and spectinomycin also showed substantial phenotypic variability, while colistin MICs remained low in most isolates. A small subset of isolates displayed elevated colistin MICs according to the applied interpretive criteria, but no molecular data were available to assess the presence of plasmid-mediated colistin resistance determinants.

**Figure 10:**
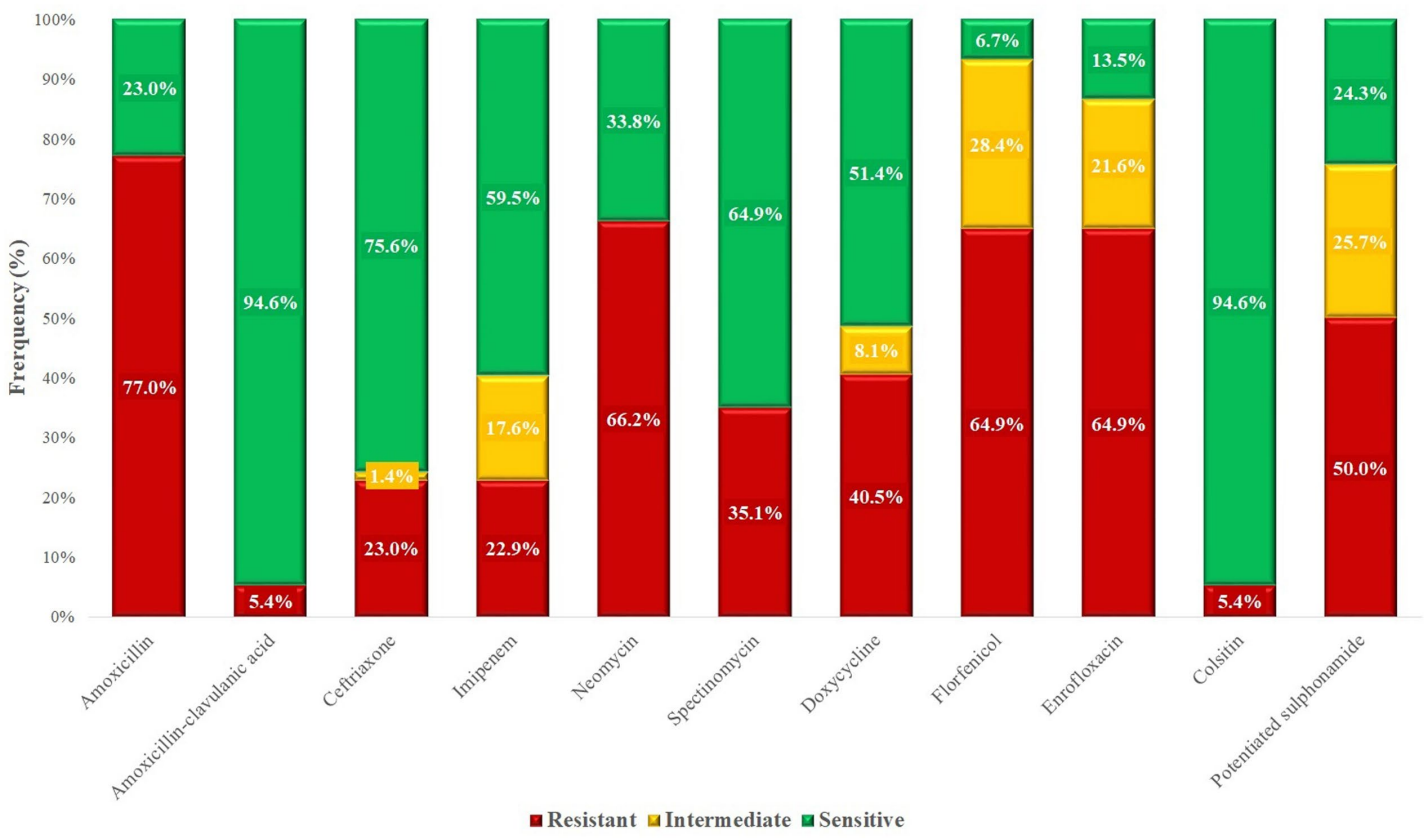
Phenotypic antimicrobial susceptibility profile of *Escherichia coli* isolates (*n* = 74) from broiler chickens in Southern Transdanubia, Hungary.

The antimicrobial susceptibility profile of *E. coli* isolates is shown in Figure 10. Reduced susceptibility was most frequent for amoxicillin (57/74, 77.0%), potentiated sulfonamides (37/74, 50.0%), florfenicol (48/74, 64.9%), enrofloxacin (48/74, 64.9%), neomycin (49/74, 66.2%), and doxycycline (30/74, 40.5%), whereas colistin reduced susceptibility was detected only in a small subset of isolates (4/74, 5.4%). These findings indicate that commensal *E. coli* isolates from the sampled chicken flocks carried a substantial burden of phenotypic reduced susceptibility within the tested antimicrobial panel.

**Figure 11:**
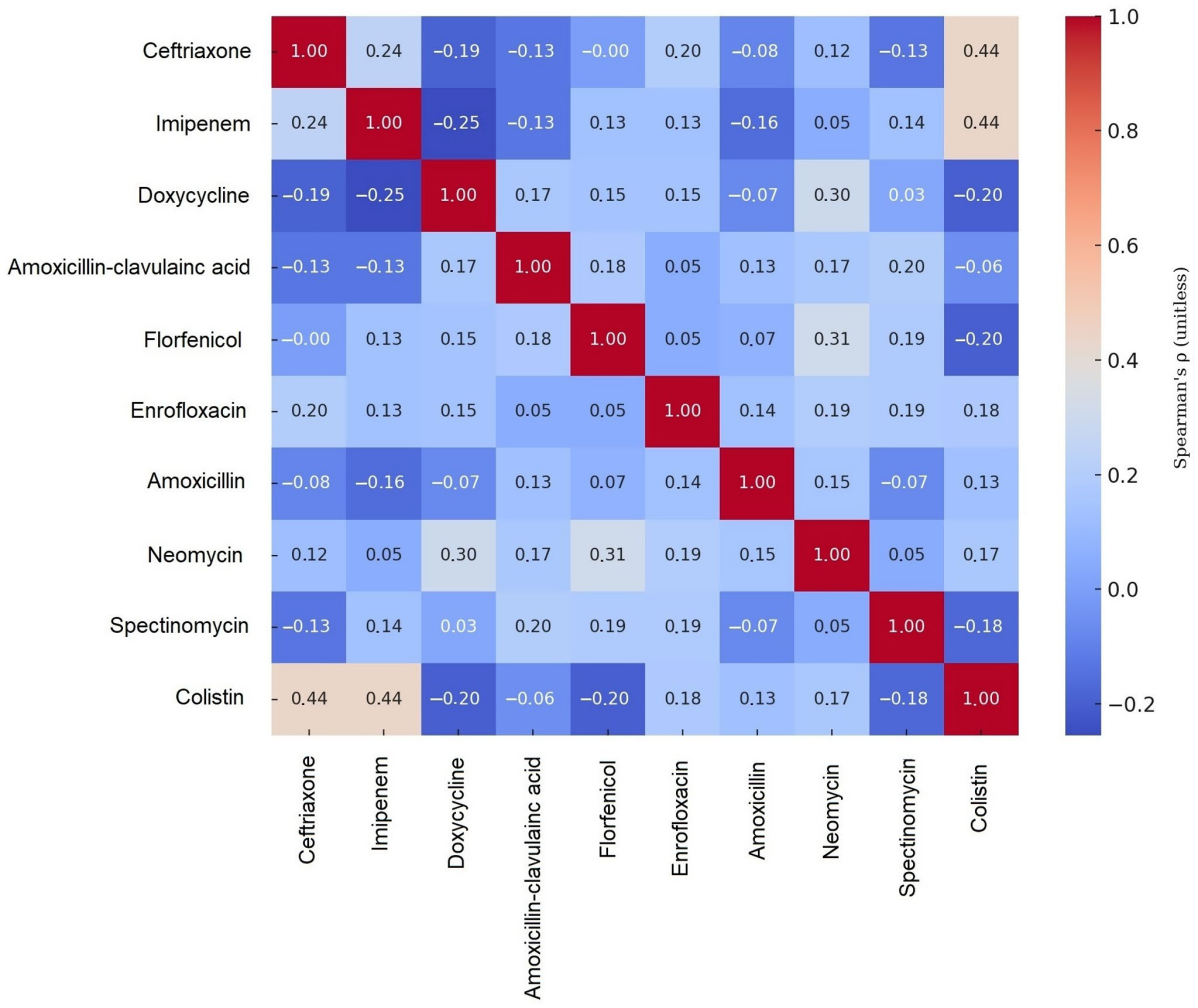
Spearman correlation heatmap of *Escherichia coli* isolates (*n* = 74) isolated from broiler chickens in the Southern Transdanubia region. Positive values indicate co-occurrence of reduced susceptibility phenotypes, values near zero suggest weak or no association, and negative values indicate inverse phenotypic associations. The heatmap was interpreted descriptively and was used to visualize exploratory co-occurrence patterns rather than to provide confirmatory statistical evidence.

Exploratory Spearman correlation analysis revealed positive associations between selected antimicrobial pairs, indicating co-occurrence of reduced susceptibility in subsets of isolates (Figure 11). The strongest phenotypic co-resistance patterns involved antimicrobial agents for which elevated MICs were frequently observed. These results suggest structured phenotypic co-resistance within the *E. coli* collection but do not allow inference of resistance-gene co-localization or horizontal transfer.

**Figure 12:**
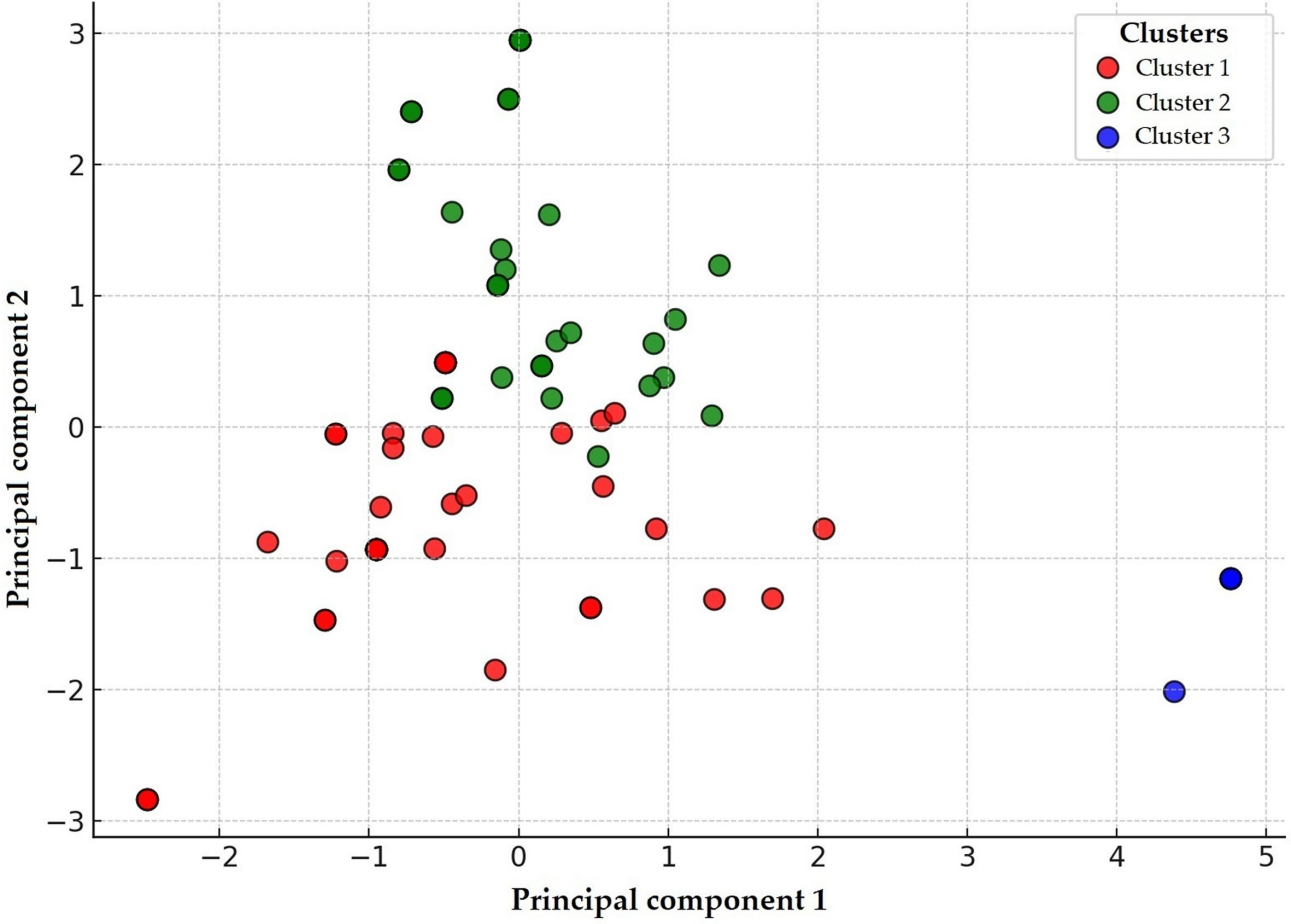
PCA and clustering of *Escherichia coli* isolates (*n =* 74) from broiler chickens in the Southern Transdanubia region. Clusters 1, 2, and 3 are represented by red, green, and blue, respectively.

Principal component analysis further supported phenotypic heterogeneity among the *E. coli* isolates (Figure 12). The distribution of isolates in the PCA space reflected differences in antimicrobial susceptibility profiles, with some isolates grouping according to broader reduced-susceptibility patterns. As with the other bacterial groups, this analysis was interpreted as exploratory and phenotype-based.

**Figure 13:**
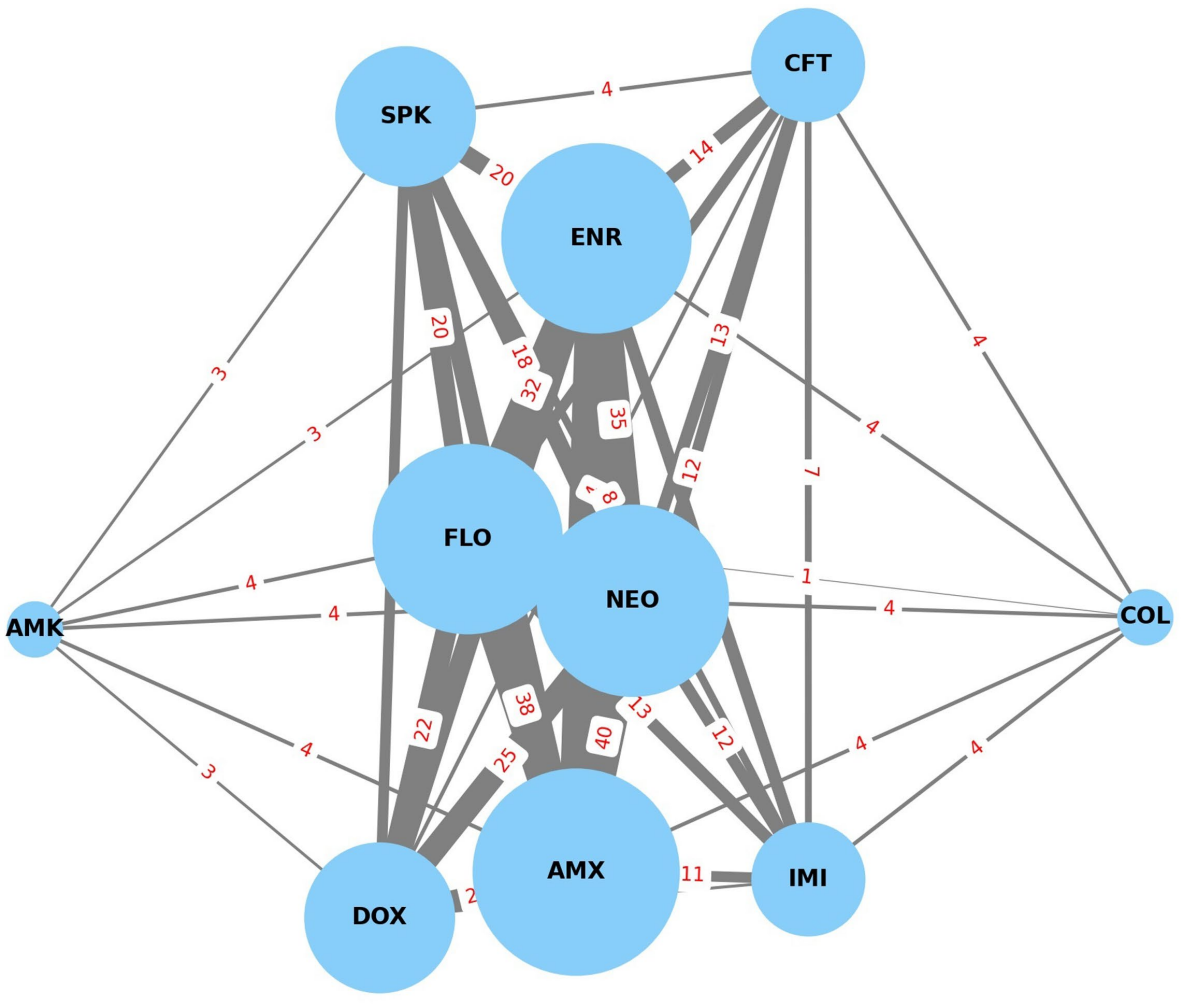
Phenotypic co-resistance network of *Escherichia coli* isolates (*n* = 74) from broiler chickens in the Southern Transdanubia region. AMK, amoxicillin-clavulanic acid; CFT, ceftriaxone; NEO, neomycin; SPK, spectinomycin; DOX, doxycycline; FLO, florfenicol; ENR, enrofloxacin; IMI, imipenem; COL, colistin.

Network-based visualization illustrated pairwise phenotypic co-resistance patterns within the *E. coli* collection (Figure 13). These patterns were interpreted as descriptive co-occurrence structures and not as evidence of resistance-gene co-localization or horizontal transfer.

Additional exploratory analyses were conducted to evaluate the structure of MDR phenotypes within the *E. coli* isolate collection. The supplementary decision tree model indicated that reduced susceptibility to selected antimicrobial agents contributed to the phenotypic classification of MDR isolates within the tested panel, although this analysis should not be interpreted as a validated predictive model (Supplementary Figure S5). A supplementary Monte Carlo simulation provided further descriptive context for the observed MDR frequency under randomized phenotype allocation and is reported as a hypothesis-generating analysis (Supplementary Figure S6).

### 3.5 Multidrug-resistant phenotypes

Multidrug-resistant phenotypes were evaluated within the limits of the tested antimicrobial panel and the available interpretive criteria. MDR was defined as reduced susceptibility or resistance to at least one antimicrobial agent in three or more antimicrobial classes among the compounds for which interpretation was possible.

MDR phenotypes were detected in 26/40 *Staphylococcus* isolates (65.0%), 62/84 *Enterococcus* isolates (73.8%), and 61/74 *E. coli* isolates (82.4%). Thus, MDR phenotypes were most frequent among *E. coli* and *Enterococcus* isolates, whereas *Staphylococcus* isolates showed a more heterogeneous distribution of susceptibility profiles. These results indicate that reduced susceptibility to multiple antimicrobial classes is not restricted to a single bacterial group within the sampled chicken-associated commensal microbiota.

Because the antimicrobial panels and available interpretive criteria differed between bacterial groups, MDR proportions should be compared cautiously across genera. The MDR analysis is best interpreted as an indicator of phenotypic burden within each bacterial group rather than as a direct quantitative comparison of resistance severity between *Staphylococcus*, *Enterococcus*, and *E. coli*. Standardized genomic and epidemiological analyses would be required to determine whether MDR phenotypes are associated with specific resistance determinants, mobile genetic elements, or farm-level antimicrobial use patterns.

An additional exploratory categorization of multidrug reduced-susceptibility burden is provided in the Supplementary material. Because the antimicrobial panels and available interpretive criteria differed between bacterial groups, these supplementary categories were not used as primary outcome measures and should be interpreted only as descriptive indicators of phenotypic resistance burden within the tested isolate collection (Supplementary Figure S7).

### 3.6 Exploratory phenotypic co-resistance patterns

Across the three bacterial groups, exploratory co-resistance analyses indicated that reduced susceptibility phenotypes were not distributed uniformly across antimicrobial agents. Instead, selected antimicrobial pairs showed repeated co-occurrence of reduced susceptibility within isolates. These patterns were visible in correlation matrices and network-based visualizations.

In *Staphylococcus* isolates, co-resistance patterns were most apparent among agents with high frequencies of reduced susceptibility, including doxycycline, amoxicillin, tiamulin, enrofloxacin, and potentiated sulfonamides. In *Enterococcus* isolates, positive phenotypic associations were observed among several compounds with broad MIC distributions, including florfenicol, tylosin, enrofloxacin, amoxicillin, and vancomycin. In *E. coli*, co-occurrence patterns involved several antimicrobial agents relevant to Gram-negative poultry-associated bacteria, including amoxicillin, neomycin, enrofloxacin, florfenicol, and potentiated sulfonamides.

These findings indicate structured phenotypic co-resistance within the isolate collection. However, because the study was based on phenotypic antimicrobial susceptibility testing alone, these associations should be interpreted as hypothesis-generating. They may reflect common selection pressures, co-exposure to antimicrobial use patterns, or the co-occurrence of resistance phenotypes, but they cannot demonstrate shared genetic mechanisms.

The correlation heatmaps were interpreted descriptively, with emphasis on the magnitude and direction of pairwise coefficients rather than on formal statistical significance testing. Therefore, the observed associations should be regarded as exploratory co-occurrence patterns within the isolate collection.

Network-based visualizations provided an additional descriptive representation of pairwise phenotypic co-resistance patterns within each bacterial group. These analyses are shown in Figures 5, 9, 13 and should be interpreted as graphical summaries of co-occurrence patterns rather than as evidence of shared resistance mechanisms or genetic linkage.

### 3.7 Contextual comparison with human resistance data

A contextual comparison was performed between poultry-associated isolates from this study and available human resistance data from Hungary. This comparison was intended to provide a broad One Health perspective rather than a direct epidemiological comparison, because the poultry and human datasets differed in sampling source, bacterial population structure, clinical context, and surveillance methodology.

For *Staphylococcus* spp., poultry-associated isolates showed higher proportions of reduced susceptibility to several antimicrobial classes than the corresponding human dataset, particularly for tetracycline-class agents, fluoroquinolones, and potentiated sulfonamides (Supplementary Figure S8). For *Enterococcus* spp., poultry-associated isolates showed detectable reduced susceptibility to multiple antimicrobial classes, whereas human *E. faecalis* and *E. faecium* data showed species-specific patterns, especially for aminopenicillins (Supplementary Figure S9). For *E. coli*, poultry-associated isolates showed higher proportions of reduced susceptibility to aminoglycosides, fluoroquinolones, and selected β-lactams than the available human comparator data (Supplementary Figure S10).

These differences should be interpreted cautiously. The comparison does not establish transmission between poultry and human bacterial populations and does not imply that resistance in one source directly explains resistance in the other. Rather, the findings highlight the importance of harmonized surveillance frameworks that allow veterinary and human AMR data to be compared using standardized sampling, testing, and interpretive approaches.

## 4 Discussion

This study provides region-specific phenotypic antimicrobial susceptibility data for chicken-associated commensal *Staphylococcus*, *Enterococcus*, and *Escherichia coli* isolates collected from large-scale flocks in Southern Transdanubia, Hungary. The results indicate that reduced susceptibility to multiple antimicrobial agents is common in commensal bacterial populations associated with intensive chicken production in this region. Although the study was based on phenotypic MIC data and did not include genomic confirmation of resistance determinants, the observed MIC distributions, MDR frequencies, and exploratory co-resistance patterns provide useful baseline information for veterinary antimicrobial resistance surveillance within a One Health framework. In settings where routine genomic surveillance remains limited, standardized MIC-based datasets such as this provide a practical basis for detecting regional phenotypic signals, prioritizing bacterial groups or antimicrobial classes for molecular follow-up, and supporting antimicrobial stewardship decisions at the production-system level.

A central strength of this study is the parallel assessment of three bacterial groups that are relevant from complementary surveillance perspectives. *E. coli* is widely used as an indicator organism for Gram-negative antimicrobial resistance in food-producing animals; enterococci are informative Gram-positive commensals with intrinsic and acquired resistance traits; and staphylococci provide additional insight into opportunistic Gram-positive bacteria associated with poultry and the food-chain environment. Interpreting these bacterial groups together allows a broader phenotypic assessment of antimicrobial susceptibility in chicken-associated commensal bacteria, while still recognizing that direct quantitative comparisons across genera must be made cautiously because antimicrobial panels, intrinsic resistance properties, and available interpretive criteria differ between bacterial groups.

Among *Staphylococcus* isolates, reduced susceptibility was frequent for several antimicrobial agents, including doxycycline, amoxicillin, potentiated sulfonamides, tiamulin, and enrofloxacin. This broad phenotypic heterogeneity is consistent with recent poultry-focused studies reporting variable antimicrobial susceptibility profiles among *Staphylococcus* spp. and *S. aureus* isolates from commercial chicken flocks, poultry meat, and related food-chain environments. In a recent Hungarian MIC-based study of commensal *Staphylococcus* spp. isolates from commercial chicken flocks, Szabó et al. (41) reported high resistance rates to tiamulin, doxycycline, and enrofloxacin, supporting the relevance of these antimicrobial classes in poultry-associated staphylococcal surveillance. Kim et al. (43) also reported resistance to penicillin-class compounds among *S. aureus* isolates from chicken meat, although at lower levels than those observed in the present isolate collection. Earlier work demonstrated that poultry meat may harbor antimicrobial-resistant *S. aureus*, but this older study is now used only as historical context rather than as the primary comparator (41, 42). These differences may reflect variation in production systems, geographic origin, antimicrobial exposure history, bacterial species composition, and interpretive criteria (43). In contrast, most isolates showed low MICs for imipenem and vancomycin. Because these agents are not used in poultry production and because interpretive criteria for commensal poultry-associated staphylococci are limited, these data are best interpreted descriptively rather than as indicators of clinical therapeutic relevance.

For *Enterococcus* isolates, MDR phenotypes and broad MIC distributions were common. This is consistent with the recognized role of enterococci as important Gram-positive commensal indicators in antimicrobial resistance surveillance. Enterococci are naturally adapted to diverse ecological niches and possess both intrinsic resistance traits and the capacity to acquire additional resistance determinants. More recent poultry-focused studies from Europe and Hungary provide a more directly relevant comparison than older geographically distant reports. In a recent Hungarian MIC-based study of poultry-associated commensal *Enterococcus* isolates, Kerek et al. (44) reported frequent reduced susceptibility to several antimicrobial classes, including tetracyclines, macrolides, pleuromutilins, fluoroquinolones, and potentiated sulfonamides. Similarly, Cagnoli et al. (20) reported antimicrobial-resistant *Enterococcus* spp. in poultry-associated samples in Italy, indicating that commensal enterococci from poultry may carry phenotypic and genotypic resistance traits even in flocks without recent antimicrobial treatment. Semedo-Lemsaddek et al. (45) found differences between free-range and conventional broiler systems, further supporting the interpretation that production system, management intensity, and antimicrobial exposure history may influence enterococcal resistance profiles.

Doxycycline reduced susceptibility among *Enterococcus* isolates was consistent with the broader observation that tetracycline-class resistance remains common in poultry-associated enterococci. In broiler breeder farms, Noh et al. (46) reported high resistance to tetracycline and doxycycline among commensal *Enterococcus faecalis* isolates, while Kerek et al. (44) also found tetracycline-class reduced susceptibility to be an important component of the phenotypic AMR profile of poultry-associated enterococci in Hungary. Schwaiger et al. (47) reported differences between organic and conventional laying-hen systems, which provides useful comparative context but should be interpreted cautiously because laying-hen production differs from the broiler-type flocks sampled in the present study. Overall, direct comparison between studies must be cautious because isolate origin, production system, antimicrobial exposure history, species composition, antimicrobial panel, and interpretive standards may differ substantially.

Florfenicol and tylosin showed notable phenotypic variability among the *Enterococcus* isolates. Previous studies have reported highly variable florfenicol resistance rates in enterococci from food-producing animals (47), ranging from very low frequencies to substantially higher levels (48) depending on the population studied (49). In the present study, the co-occurrence of reduced susceptibility to florfenicol, tylosin, and other agents was observed in exploratory analyses. These patterns may be compatible with co-selection under antimicrobial exposure, but phenotypic data alone cannot determine whether resistance determinants are genetically linked, located on mobile elements, or shared across isolates. Genomic investigation would be required to clarify whether such co-resistance patterns reflect specific resistance genes, mobile genetic elements, or independent phenotypic traits.

Tylosin reduced susceptibility was among the most frequent phenotypes observed in the present *Enterococcus* collection, exceeding the 53–63.6% range reported by Kim et al. (49). Enrofloxacin reduced susceptibility was also frequent and was broadly comparable to the high levels reported by Oliveira et al. (50), although lower rates have been described by Karunarathna et al. (48) and Liu et al. (51), who reported 29.6%.

Vancomycin MICs in the *Enterococcus* collection require careful interpretation. Elevated vancomycin MICs were observed in a subset of isolates, and similar or higher rates have been reported in some poultry-associated studies (51–53), whereas other studies detected no or only low-level vancomycin resistance (45, 47, 48, 54). Because vancomycin is a critically important antimicrobial in human medicine and is not used in poultry production, phenotypic observations involving vancomycin should not be overinterpreted without molecular confirmation. In particular, the present study did not test for *van* gene clusters or other determinants associated with acquired glycopeptide resistance. In addition, species-level interpretation is important because some enterococcal species, including *E. gallinarum* and *E. casseliflavus*, may show intrinsic low-level vancomycin resistance mediated by *vanC*, which differs substantially in epidemiological and public health significance from acquired *vanA*/*vanB*-mediated resistance. Therefore, elevated vancomycin MICs in this study may reflect different biological scenarios and should be considered a signal warranting genotype-informed investigation rather than evidence of confirmed acquired vancomycin resistance.

Among *E. coli* isolates, reduced susceptibility was frequent for several antimicrobial classes, and MDR phenotypes were particularly common. This is consistent with the role of commensal *E. coli* as a sensitive indicator of antimicrobial selection pressure in poultry production. Previous studies have reported high β-lactam resistance in poultry-associated *E. coli*, including ampicillin or amoxicillin resistance rates similar to those observed here (55, 56). Other studies, however, have reported lower rates, reflecting substantial geographic and production-system variation (57, 58). The lower MIC distribution observed for amoxicillin–clavulanic acid compared with amoxicillin alone may suggest the presence of β-lactamase-mediated phenotypes in part of the isolate collection, but this cannot be confirmed without molecular testing.

The detection of elevated ceftriaxone MICs in a subset of *E. coli* isolates is relevant from a One Health perspective because third-generation cephalosporin resistance is a major surveillance target in food-producing animals and human medicine. The proportion observed in this study was comparable to that reported by Kaushik et al. (55) in poultry-associated *E. coli*, but lower than levels reported in some other settings (59). These differences may reflect variation in antimicrobial use history, clonal expansion, biosecurity, and regional dissemination of β-lactam resistance determinants. However, because the present study did not include molecular detection of ESBL, AmpC, or other β-lactamase genes, no conclusions can be drawn regarding the underlying genetic mechanisms.

Fluoroquinolone reduced susceptibility was also common among the *E. coli* isolates. Enrofloxacin is an antimicrobial of veterinary relevance in poultry, and resistance or reduced susceptibility to fluoroquinolones in poultry-associated *E. coli* has been reported in several regions. The level observed here was higher than some previous reports (55–57, 60), but comparable to or lower than values reported in certain conventional production systems (61). These findings reinforce the importance of local surveillance, because fluoroquinolone susceptibility patterns can differ substantially across production systems and countries.

Florfenicol reduced susceptibility was frequent among *E. coli* isolates in the present study, contrasting with studies that reported low or absent florfenicol resistance in poultry-associated *E. coli* (62, 63). This discrepancy may reflect regional differences in antimicrobial use, farm-level exposure history, or differences in isolate selection and interpretation. However, without farm-level antimicrobial use data and without molecular characterization of phenicol resistance determinants, the present study cannot determine the drivers of this phenotype. The finding should therefore be interpreted as a regional phenotypic signal that merits further investigation.

Reduced susceptibility to aminoglycoside-class agents, particularly neomycin, was also common among *E. coli* isolates. Similar high levels have been reported in some poultry studies (60), whereas lower gentamicin resistance rates have been reported elsewhere (55, 57, 58). Because different aminoglycosides may have different interpretive criteria and resistance mechanisms, comparisons between neomycin and gentamicin should be made cautiously. Nevertheless, the high frequency of elevated neomycin MICs in the present collection suggests that aminoglycoside reduced susceptibility is an important component of the regional phenotypic AMR profile.

Colistin MICs remained low for most *E. coli* isolates, with only a small subset showing elevated values. This is broadly consistent with studies reporting low colistin resistance rates in poultry-associated *E. coli* (61, 62). Because colistin resistance is of high public health importance, even low-level phenotypic signals warrant attention. However, the present study did not investigate plasmid-mediated colistin resistance determinants such as *mcr* genes; therefore, the observed MIC data should not be interpreted as evidence of any specific molecular mechanism.

The exploratory co-resistance analyses indicated that reduced susceptibility phenotypes were not randomly or uniformly distributed across antimicrobial agents. In all three bacterial groups, selected antimicrobial pairs showed co-occurrence of reduced susceptibility within isolates. Such patterns are epidemiologically relevant because they may reflect common selection pressures, co-exposure to antimicrobial agents, or co-selection of phenotypes. However, this study does not provide genomic evidence for genetic linkage, resistance-gene co-localization, plasmid carriage, or horizontal gene transfer. Therefore, the co-resistance findings should be interpreted as hypothesis-generating and should guide future genomic analyses rather than serve as mechanistic conclusions.

The contextual comparison with human resistance data should also be interpreted with caution. Differences were observed between poultry-associated isolates and human datasets for several antimicrobial classes, particularly among *E. coli* and enterococci. However, these comparisons do not establish transmission between poultry and humans. The poultry isolates in this study were commensal bacteria collected from chicken flocks, whereas human datasets often reflect clinical or surveillance isolates with different sampling structures, host populations, disease contexts, and interpretive frameworks. The value of this comparison lies in highlighting the need for harmonized One Health surveillance rather than in inferring direct epidemiological linkage.

From a One Health perspective, the findings support the need for coordinated antimicrobial resistance monitoring across veterinary, food-chain, environmental, and human health sectors. Commensal bacteria from poultry can serve as useful indicators of antimicrobial selection pressure within production systems and may help identify resistance phenotypes that deserve further investigation. However, phenotypic surveillance should ideally be integrated with antimicrobial use data, farm-management information, and genomic characterization. Such integration would allow stronger inference regarding the drivers, persistence, and potential dissemination routes of antimicrobial resistance in poultry-associated bacterial populations.

This study has several limitations. First, the dataset was regional and included isolates from large-scale chicken flocks in Southern Transdanubia; therefore, the findings should not be interpreted as nationally representative estimates for Hungary. Second, the study was based on phenotypic MIC testing and did not include whole-genome sequencing or targeted molecular detection of resistance genes. As a result, no conclusions can be drawn regarding specific resistance determinants, plasmid carriage, mobile genetic elements, or clonal relationships. Third, validated clinical breakpoints or epidemiological cut-off values were not available for every organism–antimicrobial combination. For this reason, some MIC results were interpreted descriptively, and cross-compound or cross-genus comparisons should be made cautiously. Fourth, farm-level antimicrobial use data were not available, limiting the ability to link observed phenotypes to specific antimicrobial exposure histories. Finally, the exploratory statistical analyses were intended to describe phenotypic structure and co-resistance patterns within the isolate collection; they were not designed as validated predictive models or as evidence of causality. Although birds from flocks under active antimicrobial treatment at the time of sampling were not included, detailed historical antimicrobial use records were unavailable, limiting inference regarding the relationship between previous antimicrobial exposure and observed phenotypes.

Despite these limitations, the study provides useful regional baseline data on phenotypic antimicrobial susceptibility in chicken-associated commensal bacteria. The high frequency of MDR phenotypes, particularly among *E. coli* and *Enterococcus* isolates, and the presence of structured phenotypic co-resistance patterns indicate that commensal bacteria from poultry production systems remain important targets for AMR surveillance. Future studies should combine standardized MIC testing with whole-genome sequencing, farm-level antimicrobial use records, and harmonized One Health sampling frameworks to clarify the mechanisms and epidemiological relevance of the phenotypic patterns observed here.

## 5 Conclusion

This study provides region-specific phenotypic baseline data on antimicrobial susceptibility among chicken-associated commensal *Staphylococcus*, *Enterococcus*, and *Escherichia coli* isolates from large-scale flocks in Southern Transdanubia, Hungary. The findings show that reduced susceptibility to multiple antimicrobial agents is common in the sampled poultry-associated bacterial populations, with frequent MDR phenotypes particularly among *E. coli* and *Enterococcus* isolates. Exploratory co-resistance analyses further indicated structured phenotypic co-occurrence of reduced susceptibility to selected antimicrobial classes, although these patterns cannot be interpreted as evidence of shared genetic determinants or horizontal gene transfer without molecular confirmation.

Overall, the results support the value of standardized phenotypic surveillance as an essential first-line tool for monitoring antimicrobial resistance in poultry production systems. Because the study was regional and phenotype-based, the findings should be interpreted as baseline surveillance data rather than as nationally representative estimates or mechanistic evidence of resistance dissemination. Future studies should integrate MIC-based surveillance with whole-genome sequencing, farm-level antimicrobial use data, and harmonized One Health sampling frameworks to clarify the genetic background, epidemiological drivers, and potential public health relevance of the resistance patterns observed in poultry-associated commensal bacteria.

## Data Availability

The original contributions presented in the study are included in the article/Supplementary material, further inquiries can be directed to the corresponding author.
