# Supplementary material for: Regional phenotypic surveillance of antimicrobial susceptibility in chicken-associated commensal Staphylococcus, Enterococcus, and Escherichia coli from Southern Transdanubia, Hungary
Source: Front Vet Sci. 2026 Jul 2;13:1873052. doi: 10.3389/fvets.2026.1873052 (PMC13374840; doi:10.3389/fvets.2026.1873052)
Supplement: Supplementary file 1 [file Supplementary_file_1.docx]

Supplementary Material

**Supplementary Table 1.** Species identification of *Staphylococcus* isolates (*n*=40) isolated from poultry using MALDI-TOF.

| **Number** | **MALDI-TOF** | **Log (score)** |
| --- | --- | --- |
| 1 | *Staphylococcus aureus ssp. aureus* | 2.41 |
| 2 | *Staphylococcus aureus ssp. aureus* | 2.29 |
| 3 | *Staphylococcus aureus ssp. aureus* | 2.49 |
| 4 | *Staphylococcus* | 2.22 |
| 5 | *Staphylococcus aureus ssp. aureus* | 2.34 |
| 6 | *Staphylococcus aureus ssp. aureus* | 2.47 |
| 7 | *Staphylococcus aureus ssp. aureus* | 2.36 |
| 8 | *Staphylococcus aureus ssp. aureus* | 2.37 |
| 9 | *Staphylococcus aureus ssp. aureus* | 2.25 |
| 10 | *Staphylococcus gallinarum* | 2.29 |
| 11 | *Staphylococcus aureus ssp. aureus* | 2.36 |
| 12 | *Staphylococcus delphini* | 2.38 |
| 13 | *Staphylococcus aureus ssp. aureus* | 2.34 |
| 14 | *Staphylococcus gallinarum* | 2.89 |
| 15 | *Staphylococcus gallinarum* | 2.16 |
| 16 | *Staphylococcus gallinarum* | 2.44 |
| 17 | *Staphylococcus gallinarum* | 2.46 |
| 18 | *Staphylococcus delphini* | 2.47 |
| 19 | *Staphylococcus aureus ssp. aureus* | 2.31 |
| 20 | *Staphylococcus aureus ssp. aureus* | 2.36 |
| 21 | *Staphylococcus aureus ssp. aureus* | 2.58 |
| 22 | *Staphylococcus aureus ssp. aureus* | 2.74 |
| 23 | *Staphylococcus aureus ssp. aureus* | 2.46 |
| 24 | *Staphylococcus aureus ssp. aureus* | 2.56 |
| 25 | *Staphylococcus aureus ssp. aureus* | 2.39 |
| 26 | *Staphylococcus aureus ssp. aureus* | 2.31 |
| 27 | *Staphylococcus delphini* | 2.37 |
| 28 | *Staphylococcus aureus ssp. aureus* | 2.38 |
| 29 | *Staphylococcus gallinarum* | 2.41 |
| 30 | *Staphylococcus aureus ssp. aureus* | 2.44 |
| 31 | *Staphylococcus aureus ssp. aureus* | 2.46 |
| 32 | *Staphylococcus delphini* | 2.45 |
| 33 | *Staphylococcus aureus ssp. aureus* | 2.19 |
| 34 | *Staphylococcus aureus ssp. aureus* | 2.89 |
| 35 | *Staphylococcus aureus ssp. aureus* | 2.81 |
| 36 | *Staphylococcus gallinarum* | 2.55 |
| 37 | *Staphylococcus delphini* | 2.47 |
| 38 | *Staphylococcus aureus ssp. aureus* | 2.31 |
| 39 | *Staphylococcus aureus ssp. aureus* | 2.37 |
| 40 | *Staphylococcus gallinarum* | 2.33 |

2.00 – 3.00 score: High Confidence Identification

**Supplementary Table S2.** Species identification of *Enterococcus* isolates (*n*=84) isolated from poultry using MALDI-TOF.

| **Number** | **MALDI-TOF** | **Log (score)** |
| --- | --- | --- |
| 1 | *Enterococcus faecalis* | 2.39 |
| 2 | *Enterococcus faecalis* | 2.25 |
| 3 | *Enterococcus faecalis* | 2.47 |
| 4 | *Enterococcus gallinarum* | 2.41 |
| 5 | *Enterococcus gallinarum* | 2.35 |
| 6 | *Enterococcus durans* | 2.37 |
| 7 | *Enterococcus faecalis* | 2.39 |
| 8 | *Enterococcus faecium* | 2.22 |
| 9 | *Enterococcus faecium* | 2.45 |
| 10 | *Enterococcus faecium* | 2.66 |
| 11 | *Enterococcus faecium* | 2.68 |
| 12 | *Enterococcus faecalis* | 2.39 |
| 13 | *Enterococcus gallinarum* | 2.22 |
| 14 | *Enterococcus faecium* | 2.12 |
| 15 | *Enterococcus durans* | 2.14 |
| 16 | *Enterococcus faecium* | 2.19 |
| 17 | *Enterococcus faecium* | 2.55 |
| 18 | *Enterococcus faecalis* | 2.67 |
| 19 | *Enterococcus durans* | 2.79 |
| 20 | *Enterococcus durans* | 2.46 |
| 21 | *Enterococcus faecium* | 2.26 |
| 22 | *Enterococcus faecalis* | 2.21 |
| 23 | *Enterococcus faecalis* | 2.64 |
| 24 | *Enterococcus durans* | 2.49 |
| 25 | *Enterococcus hirae* | 2.39 |
| 26 | *Enterococcus durans* | 2.29 |
| 27 | *Enterococcus mundtii* | 2.33 |
| 28 | *Enterococcus durans* | 2.37 |
| 29 | *Enterococcus faecalis* | 2.65 |
| 30 | *Enterococcus durans* | 2.56 |
| 31 | *Enterococcus faecalis* | 2.87 |
| 32 | *Enterococcus faecalis* | 2.81 |
| 33 | *Enterococcus faecalis* | 2.42 |
| 34 | *Enterococcus faecalis* | 2.45 |
| 35 | *Enterococcus gallinarum* | 2.56 |
| 36 | *Enterococcus gallinarum* | 2.57 |
| 37 | *Enterococcus gallinarum* | 2.58 |
| 38 | *Enterococcus faecalis* | 2.14 |
| 39 | *Enterococcus faecium* | 2.09 |
| 40 | *Enterococcus faecium* | 2.39 |
| 41 | *Enterococcus hirae* | 2.47 |
| 42 | *Enterococcus faecium* | 2.78 |
| 43 | *Enterococcus faecalis* | 2.41 |
| 44 | *Enterococcus mundtii* | 2.57 |
| 45 | *Enterococcus faecalis* | 2.33 |
| 46 | *Enterococcus faecalis* | 2.47 |
| 47 | *Enterococcus gallinarum* | 2.51 |
| 48 | *Enterococcus faecalis* | 2.72 |
| 49 | *Enterococcus faecalis* | 2.58 |
| 50 | *Enterococcus faecalis* | 2.78 |
| 51 | *Enterococcus faecalis* | 2.41 |
| 52 | *Enterococcus faecalis* | 2.55 |
| 53 | *Enterococcus hirae* | 2.19 |
| 54 | *Enterococcus faecium* | 2.27 |
| 55 | *Enterococcus faecium* | 2.25 |
| 56 | *Enterococcus faecium* | 2.34 |
| 57 | *Enterococcus faecium* | 2.39 |
| 58 | *Enterococcus faecium* | 2.17 |
| 59 | *Enterococcus hirae* | 2.71 |
| 60 | *Enterococcus faecalis* | 2.41 |
| 61 | *Enterococcus durans* | 2.49 |
| 62 | *Enterococcus faecalis* | 2.39 |
| 63 | *Enterococcus faecalis* | 2.54 |
| 64 | *Enterococcus faecalis* | 2.32 |
| 65 | *Enterococcus faecalis* | 2.71 |
| 66 | *Enterococcus faecalis* | 2.73 |
| 67 | *Enterococcus faecalis* | 2.61 |
| 68 | *Enterococcus gallinarum* | 2.60 |
| 69 | *Enterococcus faecium* | 2.41 |
| 70 | *Enterococcus faecalis* | 2.43 |
| 71 | *Enterococcus gallinarum* | 2.55 |
| 72 | *Enterococcus gallinarum* | 2.47 |
| 73 | *Enterococcus faecalis* | 2.52 |
| 74 | *Enterococcus faecalis* | 2.31 |
| 75 | *Enterococcus faecalis* | 2.47 |
| 76 | *Enterococcus faecium* | 2.19 |
| 77 | *Enterococcus faecium* | 2.54 |
| 78 | *Enterococcus gallinarum* | 2.55 |
| 79 | *Enterococcus faecium* | 2.36 |
| 80 | *Enterococcus faecalis* | 2.71 |
| 81 | *Enterococcus faecalis* | 2.85 |
| 82 | *Enterococcus faecalis* | 2.89 |
| 83 | *Enterococcus faecalis* | 2.36 |
| 84 | *Enterococcus faecalis* | 2.54 |

2.00 – 3.00 score: High Confidence Identification

**Supplementary Table S3**. Species identification of *Escherichia coli* isolates (*n*=74) isolated from poultry using MALDI-TOF.

| **Number** | **MALDI-TOF** | **Log (score)** |
| --- | --- | --- |
| 1 | *Escherichia coli* | 2.48 |
| 2 | *Escherichia coli* | 2.36 |
| 3 | *Escherichia coli* | 2.23 |
| 4 | *Escherichia coli* | 2.96 |
| 5 | *Escherichia coli* | 2.62 |
| 6 | *Escherichia coli* | 2.55 |
| 7 | *Escherichia coli* | 2.34 |
| 8 | *Escherichia coli* | 2.16 |
| 9 | *Escherichia coli* | 2.39 |
| 10 | *Escherichia coli* | 2.25 |
| 11 | *Escherichia coli* | 2.65 |
| 12 | *Escherichia coli* | 2.34 |
| 13 | *Escherichia coli* | 2.45 |
| 14 | *Escherichia coli* | 2.58 |
| 15 | *Escherichia coli* | 2.59 |
| 16 | *Escherichia coli* | 2.34 |
| 17 | *Escherichia coli* | 2.71 |
| 18 | *Escherichia coli* | 2.74 |
| 19 | *Escherichia coli* | 2.85 |
| 20 | *Escherichia coli* | 2.65 |
| 21 | *Escherichia coli* | 2.41 |
| 22 | *Escherichia coli* | 2.25 |
| 23 | *Escherichia coli* | 2.36 |
| 24 | *Escherichia coli* | 2.65 |
| 25 | *Escherichia coli* | 2.74 |
| 26 | *Escherichia coli* | 2.85 |
| 27 | *Escherichia coli* | 2.59 |
| 28 | *Escherichia coli* | 2.47 |
| 29 | *Escherichia coli* | 2.32 |
| 30 | *Escherichia coli* | 2.31 |
| 31 | *Escherichia coli* | 2.24 |
| 32 | *Escherichia coli* | 2.16 |
| 33 | *Escherichia coli* | 2.41 |
| 34 | *Escherichia coli* | 2.36 |
| 35 | *Escherichia coli* | 2.15 |
| 36 | *Escherichia coli* | 2.17 |
| 37 | *Escherichia coli* | 2.54 |
| 38 | *Escherichia coli* | 2.89 |
| 39 | *Escherichia coli* | 2.91 |
| 40 | *Escherichia coli* | 2.96 |
| 41 | *Escherichia coli* | 2.19 |
| 42 | *Escherichia coli* | 2.24 |
| 43 | *Escherichia coli* | 2.34 |
| 44 | *Escherichia coli* | 2.54 |
| 45 | *Escherichia coli* | 2.19 |
| 46 | *Escherichia coli* | 2.45 |
| 47 | *Escherichia coli* | 2.59 |
| 48 | *Escherichia coli* | 2.34 |
| 49 | *Escherichia coli* | 2.16 |
| 50 | *Escherichia coli* | 2.51 |
| 51 | *Escherichia coli* | 2.60 |
| 52 | *Escherichia coli* | 2.54 |
| 53 | *Escherichia coli* | 2.19 |
| 54 | *Escherichia coli* | 2.45 |
| 55 | *Escherichia coli* | 2.49 |
| 56 | *Escherichia coli* | 2.39 |
| 57 | *Escherichia coli* | 2.47 |
| 58 | *Escherichia coli* | 2.70 |
| 59 | *Escherichia coli* | 2.45 |
| 60 | *Escherichia coli* | 2.62 |
| 61 | *Escherichia coli* | 2.79 |
| 62 | *Escherichia coli* | 2.16 |
| 63 | *Escherichia coli* | 2.14 |
| 64 | *Escherichia coli* | 2.16 |
| 65 | *Escherichia coli* | 2.47 |
| 66 | *Escherichia coli* | 2.58 |
| 67 | *Escherichia coli* | 2.64 |
| 68 | *Escherichia coli* | 2.78 |
| 69 | *Escherichia coli* | 2.22 |
| 70 | *Escherichia coli* | 2.36 |
| 71 | *Escherichia coli* | 2.11 |
| 72 | *Escherichia coli* | 2.09 |
| 73 | *Escherichia coli* | 2.27 |
| 74 | *Escherichia coli* | 2.48 |

2.00 – 3.00 score: High Confidence Identification

**
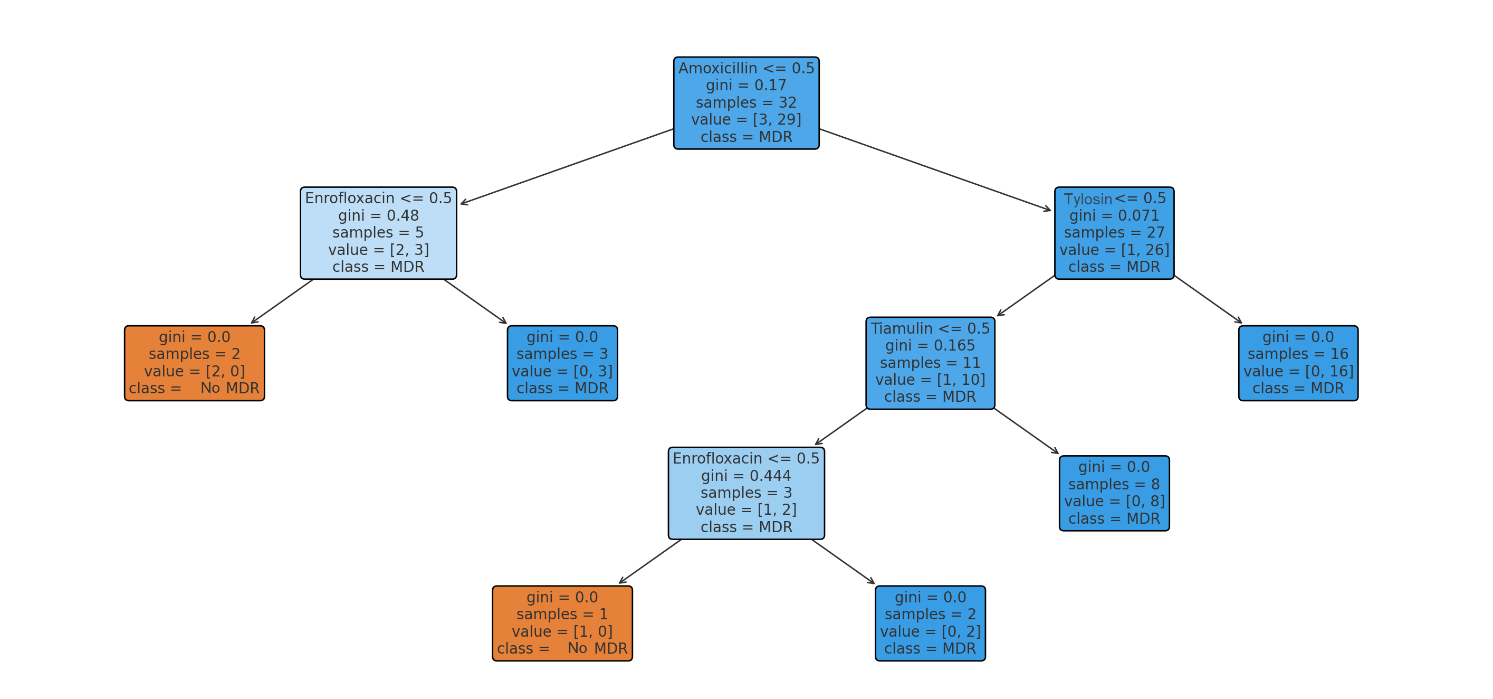
**

**Supplementary Figure S1.** Decision tree analysis of phenotypic multidrug resistance (MDR) classification in *Staphylococcus* isolates.


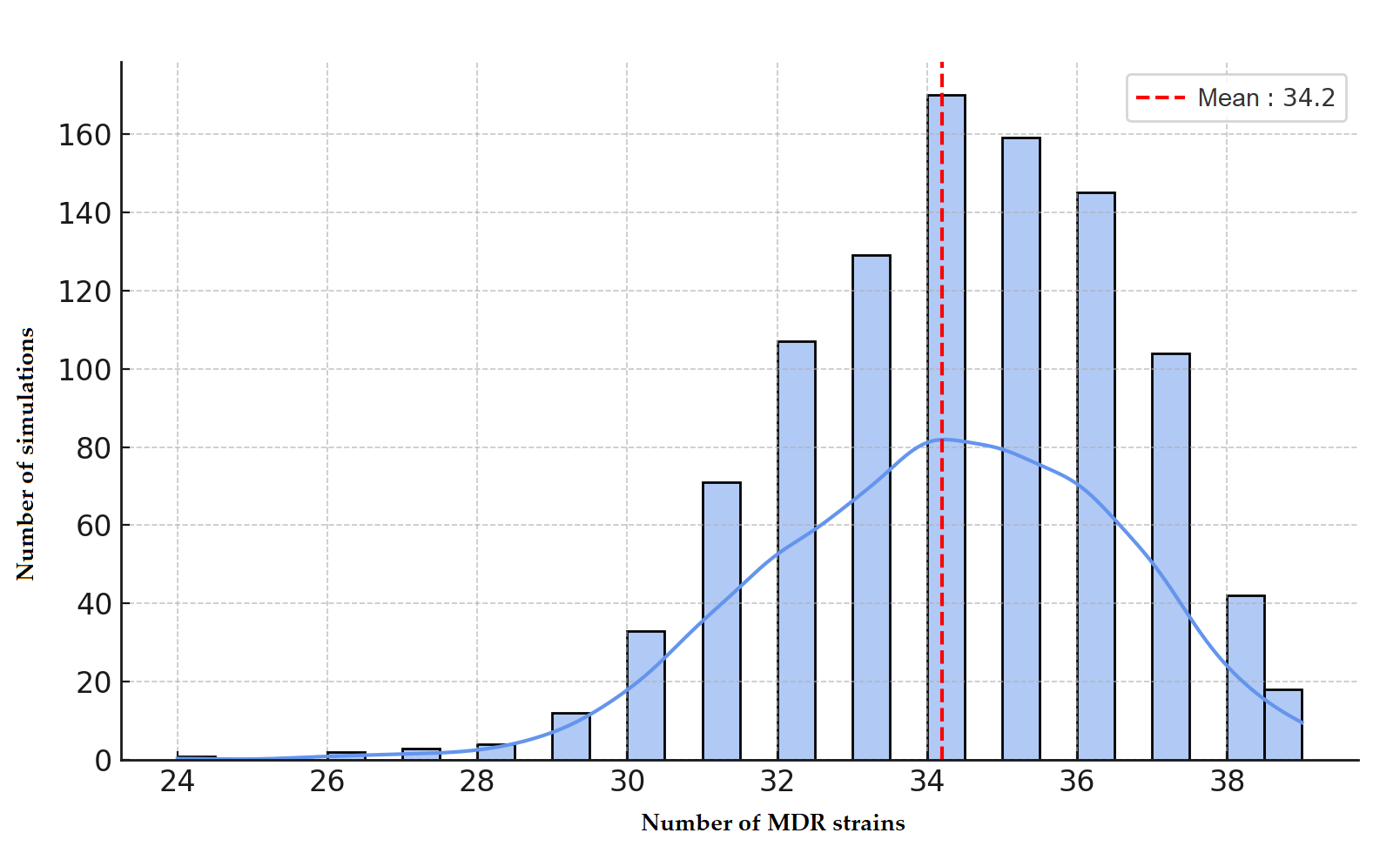


**Supplementary Figure S2.** Monte Carlo simulation of multidrug resistance (MDR) frequency in *Staphylococcus* isolates.


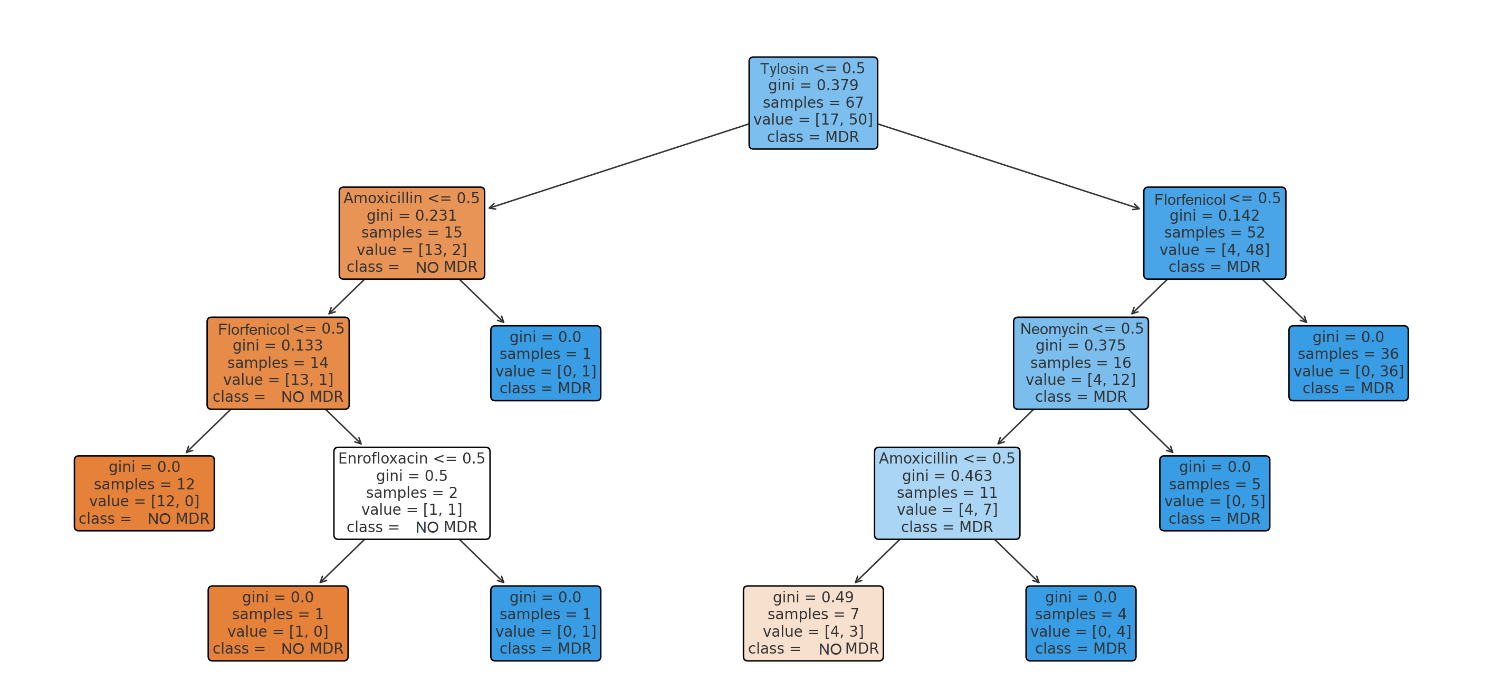


**Supplementary Figure S3.** Decision tree analysis of phenotypic multidrug resistance (MDR) classification in *Enterococcus* isolates.


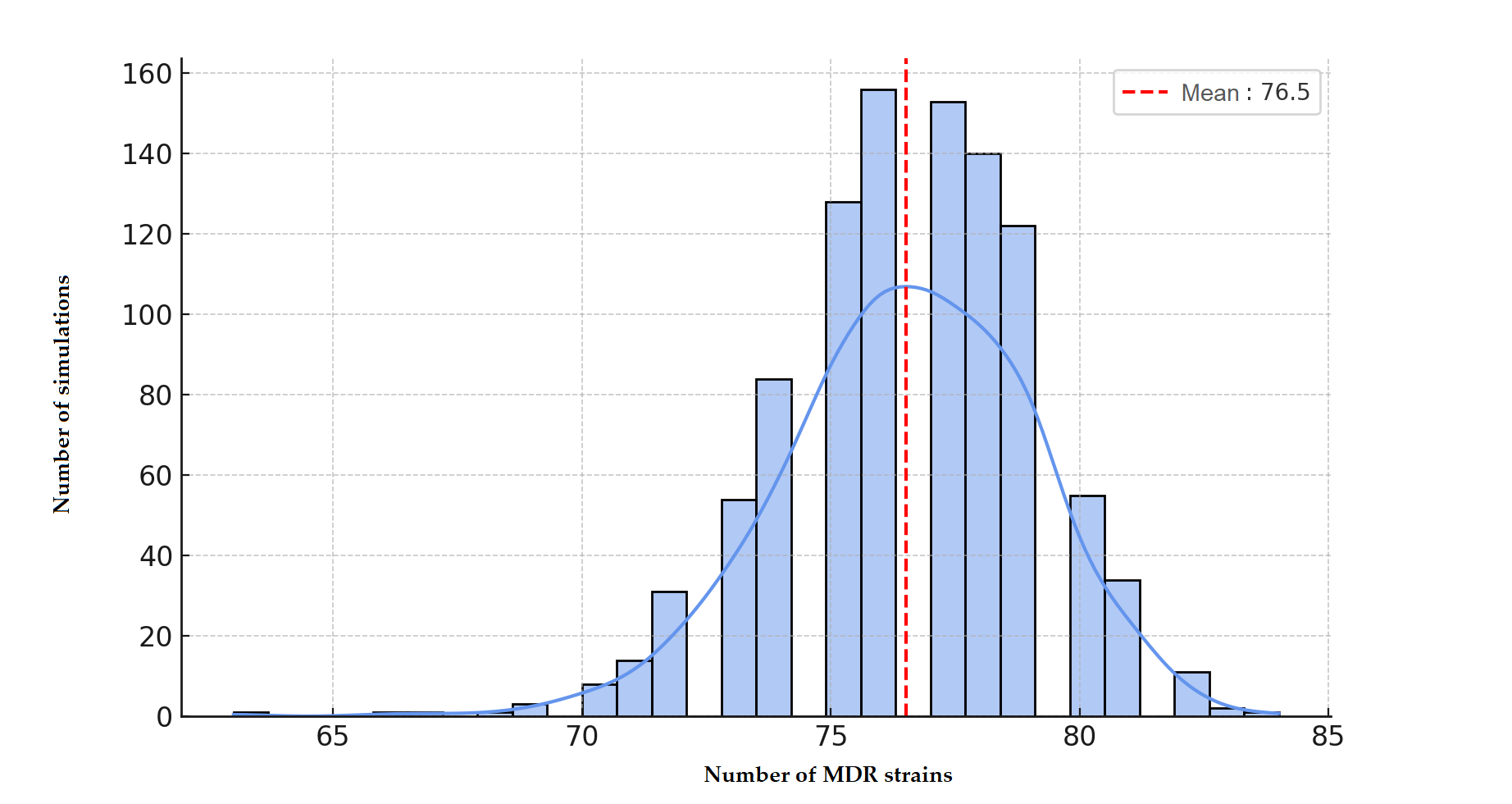


**Supplementary Figure S4.** Monte Carlo simulation of multidrug resistance (MDR) frequency in *Enterococcus* isolates.


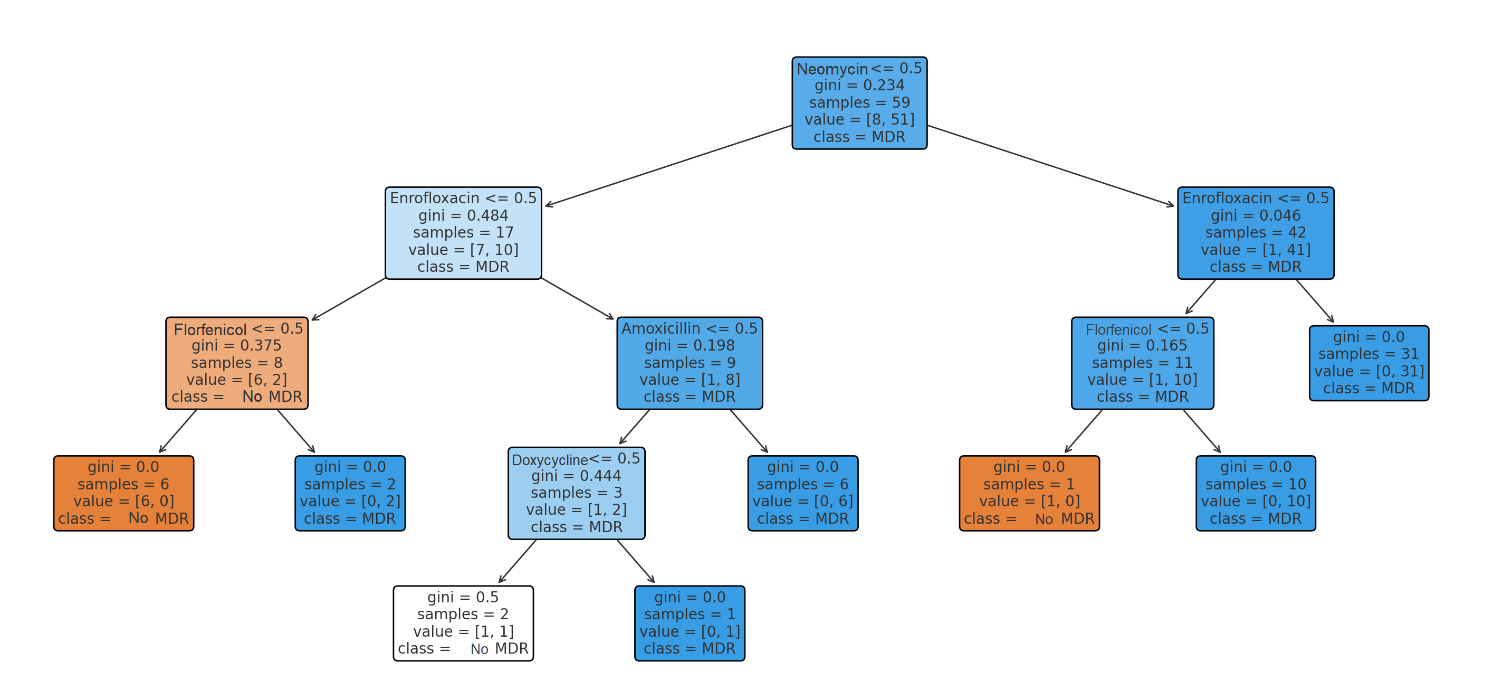


**Supplementary Figure S5.** Decision tree analysis of phenotypic multidrug resistance (MDR) classification in *Escherichia coli* isolates.


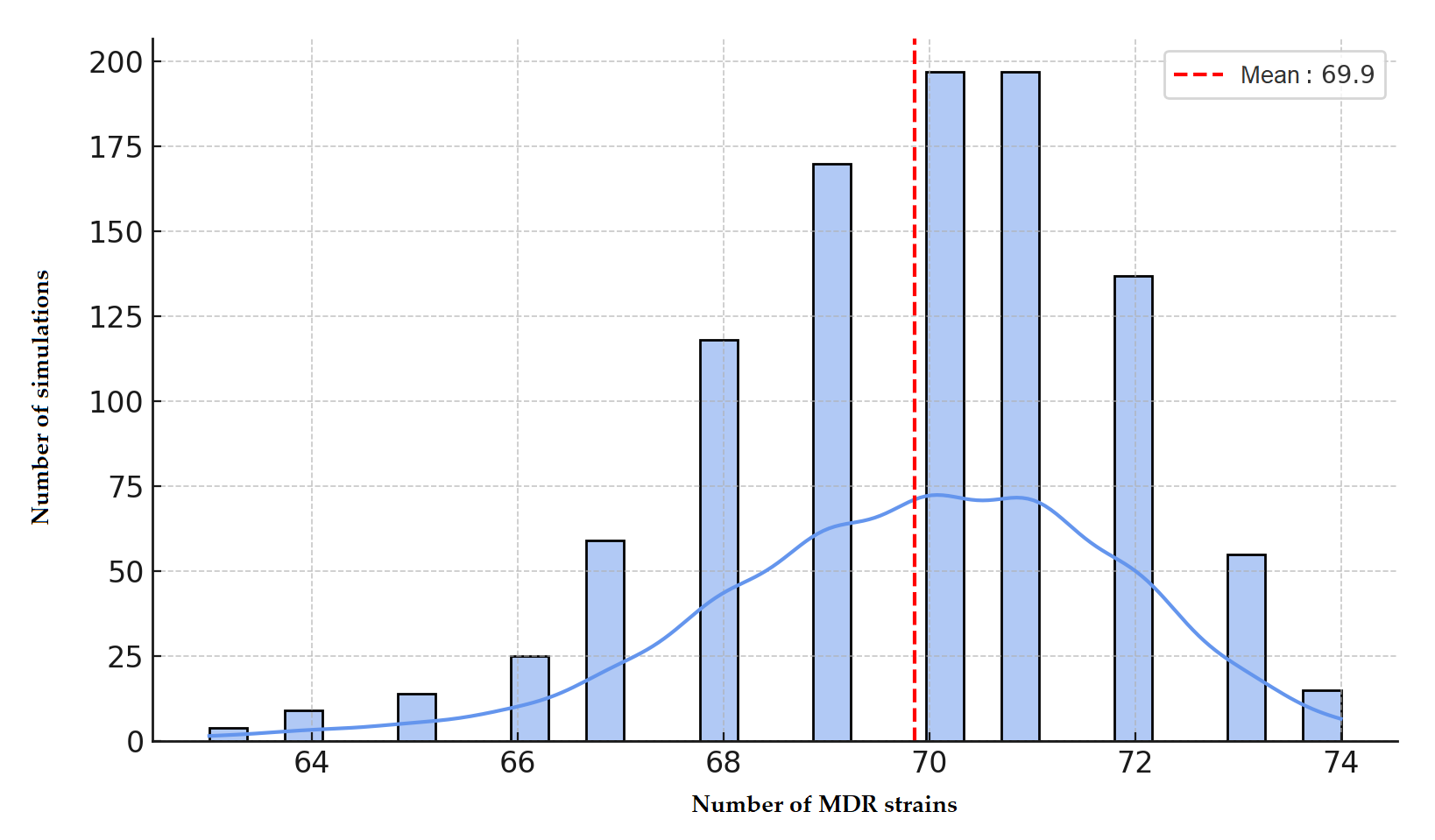


**Supplementary Figure S6.** Monte Carlo simulation of multidrug resistance (MDR) frequency in *Escherichia coli* isolates.


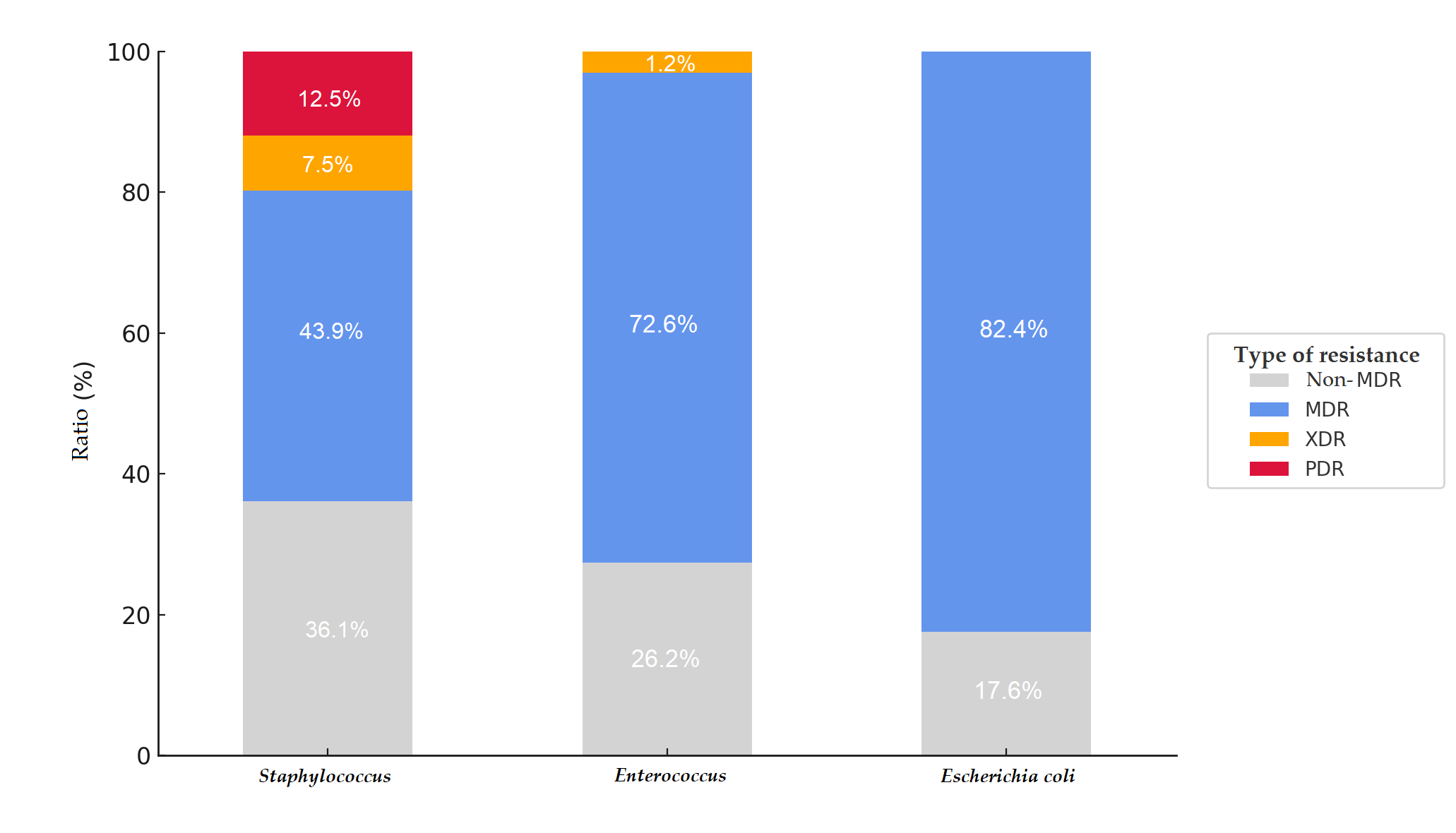


**Supplementary Figure S7.** Exploratory distribution of multidrug resistance (MDR), putative extensively drug-resistant (XDR), and putative pandrug-resistant (PDR) phenotypes across bacterial groups.


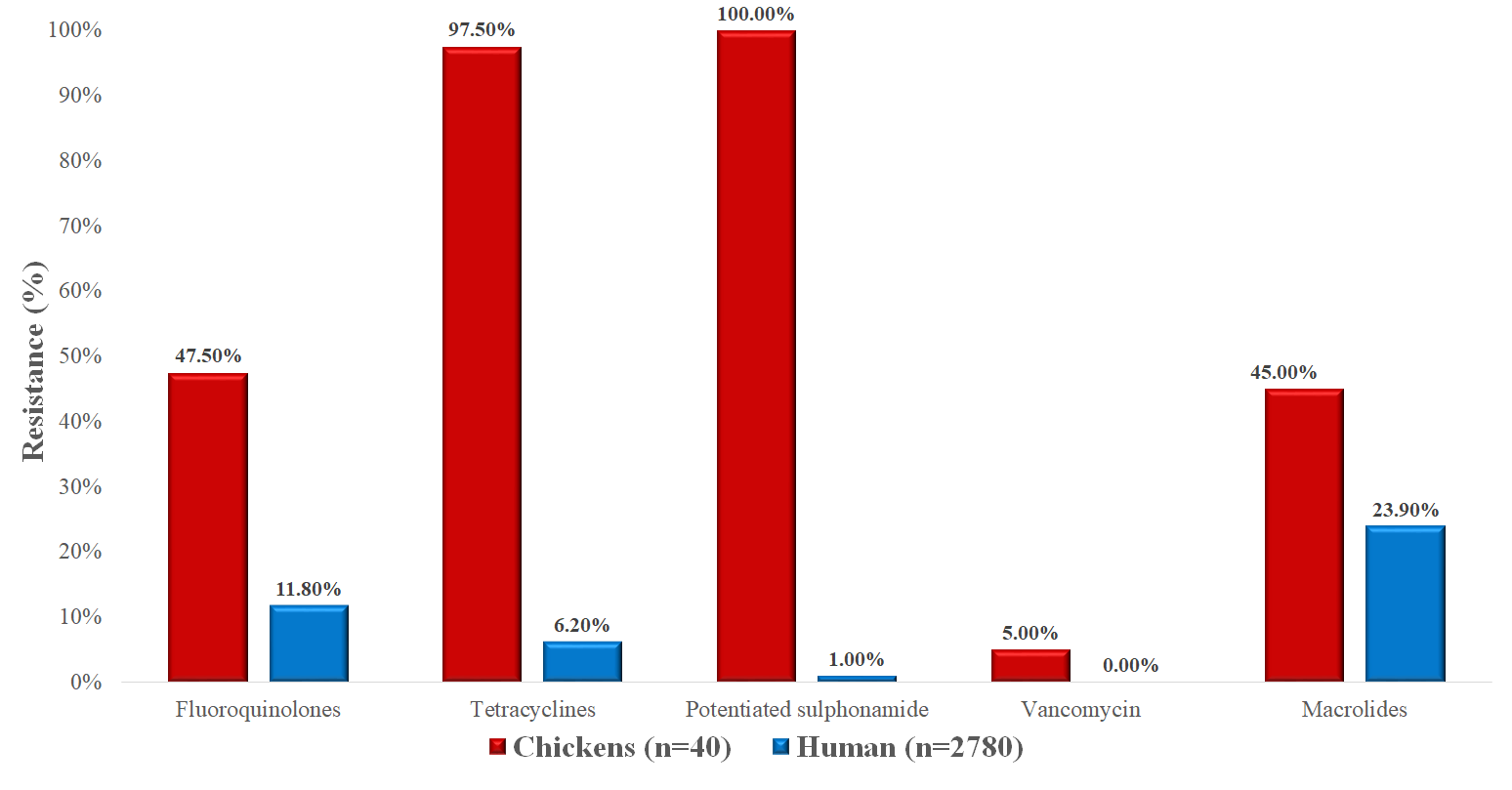


**Supplementary Figure S8.** Phenotypic co-resistance network of *Staphylococcus* isolates.


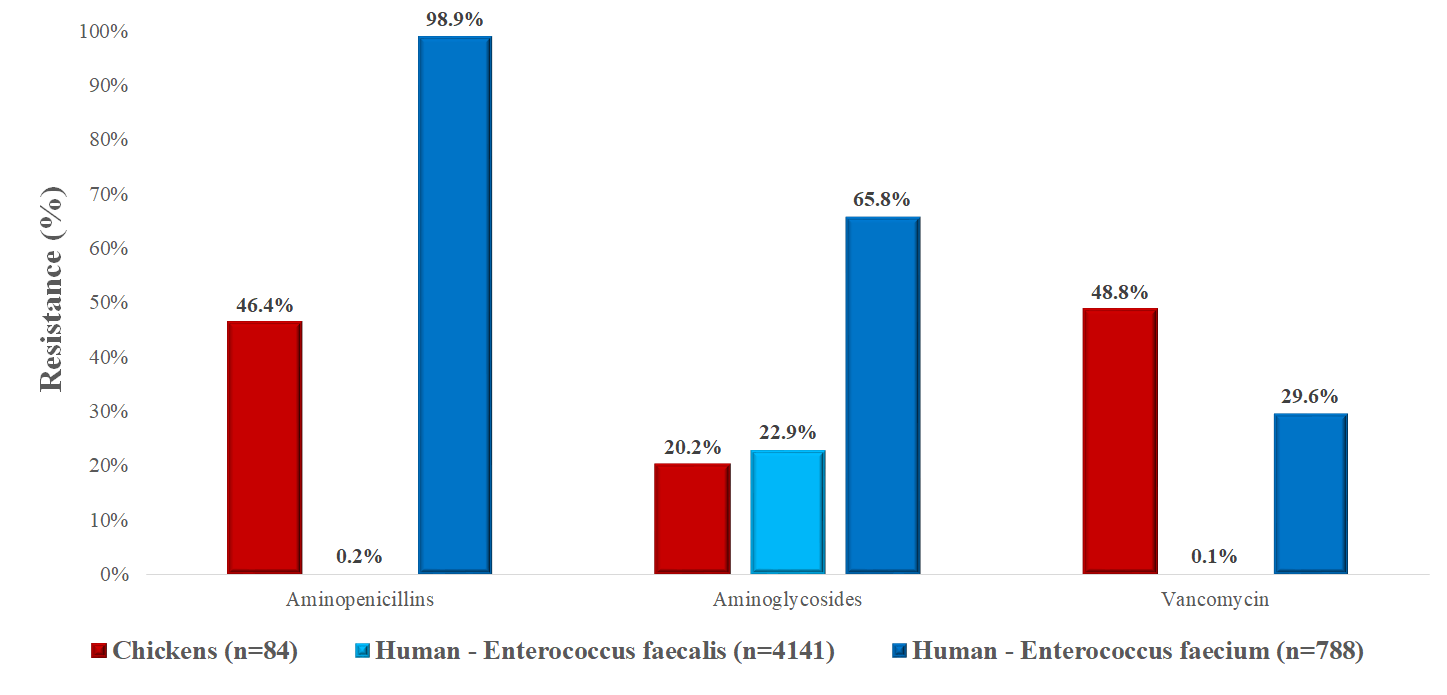


**Supplementary Figure S9.** Phenotypic co-resistance network of *Enterococcus* isolates.


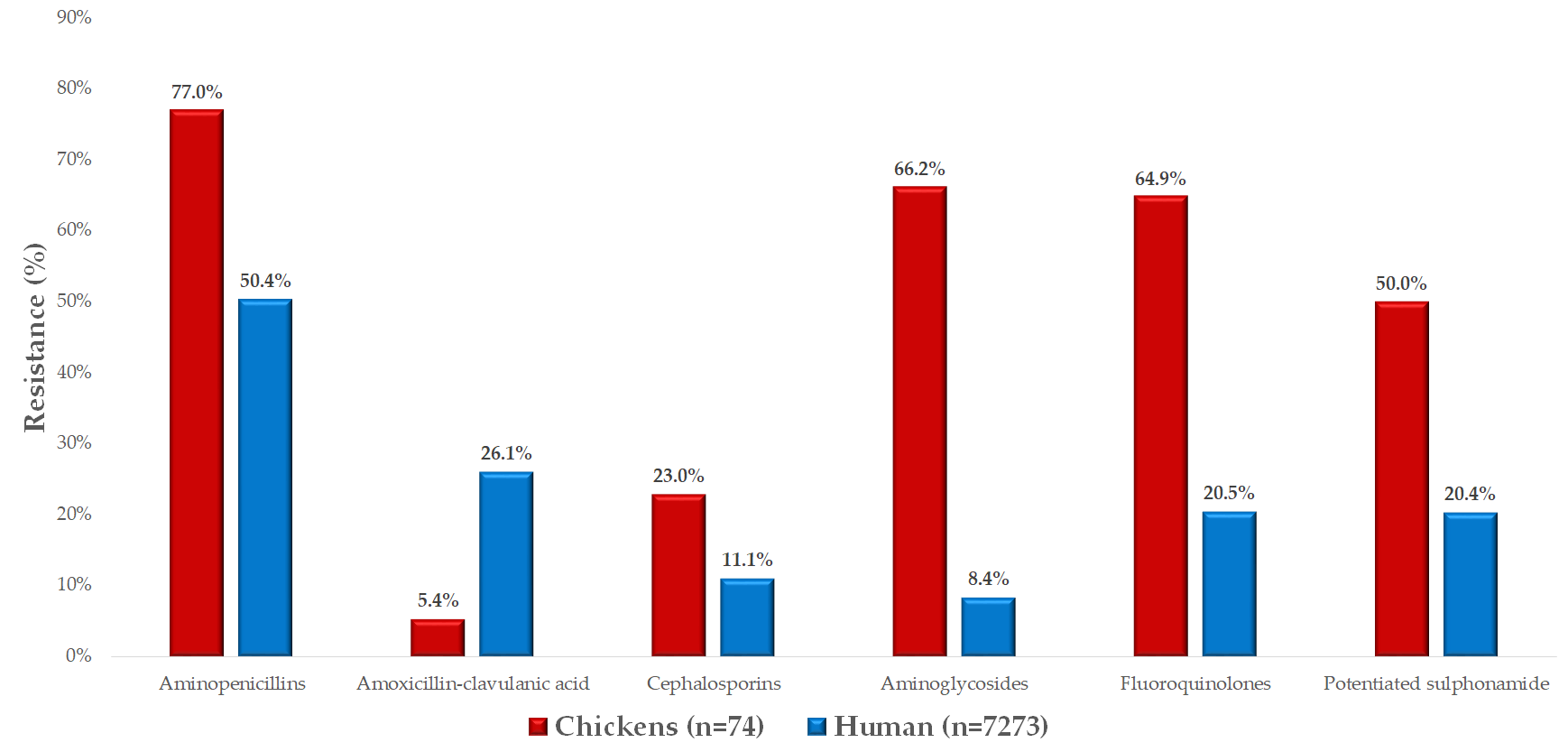


**Supplementary Figure S10.** Phenotypic co-resistance network of *Escherichia coli* isolates.

**Supplementary Table S4** Frequency distribution of minimum inhibitory concentration (MIC) values for antimicrobial agents tested against *Staphylococcus* isolates (*n*=40) from broiler chickens in the Southern Transdanubia region. The upper row for each compound displays the number of isolates, while the lower row shows the corresponding percentage. Interpretive thresholds are shown where applicable, vertical red lines indicate the clinical breakpoint, and green lines represent the epidemiological cutoff value (ECOFF).

| **Antibiotics** | **^1^Breakpoint** | **0.001** | **0.002** | **0.004** | **0.008** | **0.016** | **0.031** | **0.063** | **0.125** | **0.25** | **0.5** | **1** | **2** | **4** | **8** | **16** | **32** | **64** | **128** | **256** | **512** | **1024** | **MIC_50_** | **MIC_90_** | **^2^ECOFF** | |
| --- | --- | --- | --- | --- | --- | --- | --- | --- | --- | --- | --- | --- | --- | --- | --- | --- | --- | --- | --- | --- | --- | --- | --- | --- | --- | --- |
|  | **µg/mL** | | | | | | | | | | | | | | | | | | | | | | | | |  |
| Amoxicillin | 0.5 |  |  |  |  |  |  | 1 | 3 | 3 | 26 | 1 | 2 | 0 | 0 | 2 | 0 | 0 | 0 | 1 | 1 |  | 0.5 | 2 | 0.5 | |
|  |  |  |  |  |  |  |  | 2.5% | 7.5% | 7.5% | 65.0% | 2.5% | 5.0% | 0.0% | 0.0% | 5.0% | 0.0% | 0.0% | 0.0% | 2.5% | 2.5% |  |  |  |  |  |
| ^3^Amoxicillin-clavulanic acid | 1 |  |  |  |  |  |  | 1 | 1 | 19 | 9 | 6 | 0 | 0 | 2 | 2 |  |  |  |  |  |  | 0.25 | 1 | 0.5 | |
|  |  |  |  |  |  |  |  | 2.5% | 2.5% | 47.5% | 22.5% | 15.0% | 15.0% | 0.0% | 5.0% | 5.0% |  |  |  |  |  |  |  |  |  |  |
| Doxycycline | 0.5 |  |  |  |  |  |  | 1 | 0 | 0 | 8 | 12 | 0 | 3 | 3 | 2 | 0 | 10 | 1 |  |  |  | 0.5 | 128 | 0.5 | |
|  |  |  |  |  |  |  |  | 2.5% | 0.0% | 0.0% | 20.0% | 30.0% | 0.0% | 7.5% | 7.5% | 5.0% | 0.0% | 25.0% | 2.5% |  |  |  |  |  |  |  |
| Enrofloxacin | 4 |  |  |  |  |  |  | 1 | 0 | 0 | 0 | 19 | 19 | 7 | 4 | 1 | 2 | 2 | 3 |  |  |  | 8 | 64 | 0.5 | |
|  |  |  |  |  |  |  |  | 2.5% | 0.0% | 0.0% | 0.0% | 47.5% | 47.5% | 17.5% | 10.0% | 2.5% | 5.0% | 5.0% | 7.5% |  |  |  |  |  |  |  |
| Imipenem | 8 |  |  |  |  |  | 3 | 6 | 8 | 3 | 2 | 2 | 2 | 13 | 1 |  |  |  |  |  |  |  | 0.25 | 1 | 0.125 | |
|  |  |  |  |  |  |  | 7.5% | 15.0% | 20.0% | 7.5% | 5.0% | 5.0% | 5.0% | 32.5% | 2.5% |  |  |  |  |  |  |  |  |  |  |  |
| ^4^Potentiated sulfonamide | 4 |  |  |  |  |  |  |  |  |  |  |  |  |  | 5 | 18 | 3 | 1 | 3 | 5 | 1 | 4 | 64 | 1024 | 0.25 | |
|  |  |  |  |  |  |  |  |  |  |  |  |  |  |  | 12.5% | 45.0% | 7.5% | 2.5% | 7.5% | 12.5% | 2.5% | 10.0% |  |  |  |  |
| Tylosin | 64 |  |  |  |  |  |  |  |  |  |  | 19 | 1 | 0 | 1 | 0 | 1 | 0 | 1 | 1 | 2 | 14 | 4 | 1024 | 2 | |
|  |  |  |  |  |  |  |  |  |  |  |  | 47.5% | 2.5% | 0.0% | 2.5% | 0.0% | 2.5% | 0.0% | 2.5% | 2.5% | 5.0% | 35.0% |  |  |  |  |
| Tiamulin | 4 |  |  |  |  |  |  |  |  |  |  | 16 | 0 | 2 | 0 | 2 | 9 | 5 | 2 | 3 | 0 | 1 | 16 | 64 | 2 | |
|  |  |  |  |  |  |  |  |  |  |  |  | 40.0% | 0.0% | 5.0% | 0.0% | 5.0% | 22.5% | 12.5% | 5.0% | 7.5% | 0.0% | 2.5% |  |  |  |  |
| Vancomycin | 32 |  |  |  |  |  |  |  |  | 11 | 13 | 11 | 1 | 2 | 0 | 0 | 0 | 0 | 0 | 2 |  |  | 0.5 | 2 | 2 | |
|  |  |  |  |  |  |  |  |  |  | 27.5% | 32.5% | 27.5% | 2.5% | 5.0% | 0.0% | 0.0% | 0.0% | 0.0% | 0.0% | 5.0% |  |  |  |  |  |  |

^1^ Clinical Laboratory Standards Institute (CLSI); ^2^Epidemiological cut-off value (EUCAST); ^3^2:1 ratio; ^4^trimethoprim-sulfamethoxazole in ratio 1:19

**Supplementary Table S5.** Frequency distribution of minimum inhibitory concentration (MIC) values for antimicrobial agents tested against *Enterococcus* isolates (*n*=84) from chickens in the Southern Transdanubia region. The upper row for each compound displays the number of isolates, while the lower row shows the corresponding percentage. Interpretive thresholds are shown where applicable, vertical red lines indicate the clinical breakpoint, and green lines represent the epidemiological cutoff value (ECOFF).

| **Antibiotics** | **^1^Breakpoint** | **0.001** | **0.002** | **0.004** | **0.008** | **0.016** | **0.031** | **0.063** | **0.125** | **0.25** | **0.5** | **1** | **2** | **4** | **8** | **16** | **32** | **64** | **128** | **256** | **512** | **1024** | **MIC_50_** | **MIC_90_** | **^2^ECOFF** |
| --- | --- | --- | --- | --- | --- | --- | --- | --- | --- | --- | --- | --- | --- | --- | --- | --- | --- | --- | --- | --- | --- | --- | --- | --- | --- |
|  | **µg/mL** | | | | | | | | | | | | | | | | | | | | | | | | |
| Amoxicillin | 16 |  |  |  |  | 1 | 2 | 0 | 1 | 1 | 10 | 20 | 7 | 1 | 2 | 0 | 3 | 1 | 1 | 10 | 15 | 9 | 2 | 512 | - |
|  |  |  |  |  |  | 1.2% | 2.4% | 0.0% | 1.2% | 1.2% | 11.9% | 23.8% | 8.3% | 1.2% | 2.4% | 0.0% | 3.6% | 1.2% | 1.2% | 11.9% | 17.9% | 10.7% |  |  |  |
| ^3^Amoxicillin-clavulanic acid | 16 |  |  |  |  | 1 | 2 | 0 | 0 | 5 | 16 | 8 | 8 | 12 | 18 | 7 | 7 |  |  |  |  |  | 4 | 16 | - |
|  |  |  |  |  |  | 1.2% | 2.4% | 0.0% | 0.0% | 6.0% | 19.0% | 9.5% | 9.5% | 14.3% | 21.4% | 8.3% | 8.3% |  |  |  |  |  |  |  |  |
| Doxycycline | 16 |  |  |  |  |  |  |  |  | 1 | 1 | 13 | 4 | 16 | 15 | 13 | 10 | 10 | 1 |  |  |  | 8 | 64 | 1 |
|  |  |  |  |  |  |  |  |  |  | 1.2% | 1.2% | 15.5% | 4.8% | 19.0% | 17.9% | 15.5% | 11.9% | 11.9% | 1.2% |  |  |  |  |  |  |
| Enrofloxacin | 4 |  |  |  |  |  |  |  |  |  | 4 | 10 | 4 | 7 | 13 | 13 | 5 | 16 | 9 | 2 |  |  | 16 | 128 | - |
|  |  |  |  |  |  |  |  |  |  |  | 4.8% | 11.9% | 4.8% | 8.3% | 15.5% | 15.5% | 6.0% | 19.0% | 10.7% | 2.4% |  |  |  |  |  |
| Florfenicol | 8 |  |  |  |  |  |  |  |  |  |  |  | 12 | 26 | 26 | 8 | 7 | 2 | 2 | 1 |  |  | 8 | 32 | 8 |
|  |  |  |  |  |  |  |  |  |  |  |  |  | 14.3% | 31.0% | 31.0% | 9.5% | 8.3% | 2.4% | 2.4% | 1.2% |  |  |  |  |  |
| Imipenem | 16 |  |  |  |  | 8 | 2 | 5 | 1 | 2 | 1 | 7 | 16 | 22 | 10 | 9 | 0 | 0 | 0 | 1 |  |  | 2 | 16 | 4 |
|  |  |  |  |  |  | 9.5% | 2.4% | 6.0% | 1.2% | 2.4% | 1.2% | 8.3% | 19.0% | 26.2% | 11.9% | 10.7% | 0.0% | 0.0% | 0.0% | 1.2% |  |  |  |  |  |
| Neomycin | 1024 |  |  |  |  |  |  |  |  |  |  | 2 | 2 | 1 | 0 | 2 | 5 | 9 | 14 | 10 | 22 | 17 | 256 | 1024 | 256 |
|  |  |  |  |  |  |  |  |  |  |  |  | 2.4% | 2.4% | 1.2% | 0.0% | 2.4% | 6.0% | 10.7% | 16.7% | 11.9% | 26.2% | 20.2% |  |  |  |
| ^4^Potentiated sulfonamide | 64 |  |  |  |  |  |  |  |  |  |  | 2 | 4 | 10 | 12 | 13 | 3 | 2 | 10 | 1 | 7 | 20 | 32 | 1024 | - |
|  |  |  |  |  |  |  |  |  |  |  |  | 2.4% | 4.8% | 11.9% | 14.3% | 15.5% | 3.6% | 2.4% | 11.9% | 1.2% | 8.3% | 23.8% |  |  |  |
| Tylosin | 8 |  |  |  |  |  |  |  |  |  |  | 17 | 4 | 0 | 1 | 2 | 0 | 1 | 2 | 7 | 3 | 47 | 1024 | 1024 | - |
|  |  |  |  |  |  |  |  |  |  |  |  | 20.2% | 4.8% | 0.0% | 1.2% | 2.4% | 0.0% | 1.2% | 2.4% | 8.3% | 3.6% | 56.0% |  |  |  |
| Vancomycin | 32 |  |  |  |  |  |  |  |  |  | 13 | 17 | 5 | 4 | 3 | 1 | 0 | 1 | 7 | 7 | 1 | 25 | 8 | 1024 | 4 |
|  |  |  |  |  |  |  |  |  |  |  | 15.5% | 20.2% | 6.0% | 4.8% | 3.6% | 1.2% | 0.0% | 1.2% | 8.3% | 8.3% | 1.2% | 29.8% |  |  |  |

^1^ Clinical Laboratory Standards Institute (CLSI); ^2^Epidemiological cut-off value (EUCAST); ^3^2:1 ratio; ^4^trimethoprim-sulfamethoxazole in ratio 1:19.

**Supplementary Table S6.** Frequency distribution of minimum inhibitory concentration (MIC) values for antimicrobial agents tested against *Escherichia coli isolates* (*n*=74) from broiler chickens in the Southern Transdanubia region. The upper row for each compound displays the number of isolates, while the lower row shows the corresponding percentage. Interpretive thresholds are shown where applicable, vertical red lines indicate the clinical breakpoint, and green lines represent the epidemiological cutoff value (ECOFF).

| **Antibiotics** | **^1^Breakpoint** | **0.001** | **0.002** | **0.004** | **0.008** | **0.016** | **0.031** | **0.063** | **0.125** | **0.25** | **0.5** | **1** | **2** | **4** | **8** | **16** | **32** | **64** | **128** | **256** | **512** | **1024** | **MIC_50_** | **MIC_90_** | **^2^ECOFF** |
| --- | --- | --- | --- | --- | --- | --- | --- | --- | --- | --- | --- | --- | --- | --- | --- | --- | --- | --- | --- | --- | --- | --- | --- | --- | --- |
|  | **µg/mL** | | | | | | | | | | | | | | | | | | | | | | | | |
| Amoxicillin | 32 |  |  |  |  |  |  |  |  |  | 1 | 0 | 6 | 6 | 4 | 0 | 0 | 2 | 5 | 8 | 19 | 23 | 512 | 1024 | 8 |
|  |  |  |  |  |  |  |  |  |  |  | 1.4% | 0.0% | 8.1% | 8.1% | 5.4% | 0.0% | 0.0% | 2.7% | 6.8% | 10.8% | 25.7% | 31.1% |  |  |  |
| ^3^Amoxicillin-clavulanic acid | 32 |  |  |  |  |  |  |  |  |  |  | 1 | 3 | 13 | 22 | 31 | 1 | 3 |  |  |  |  | 8 | 16 | 8 |
|  |  |  |  |  |  |  |  |  |  |  |  | 1.4% | 4.1% | 17.6% | 29.7% | 41.9% | 1.4% | 4.1% |  |  |  |  |  |  |  |
| Ceftriaxone | 4 |  |  |  |  | 3 | 15 | 21 | 9 | 2 | 4 | 2 | 1 | 0 | 1 | 1 | 3 | 1 | 0 | 6 | 0 | 5 | 0.063 | 256 | 0.125 |
|  |  |  |  |  |  | 4.1% | 20.3% | 28.4% | 12.2% | 2.7% | 5.4% | 2.7% | 1.4% | 0.0% | 1.4% | 1.4% | 4.1% | 1.4% | 0.0% | 8.1% | 0.0% | 6.8% |  |  |  |
| Doxycycline | 16 |  |  |  |  |  |  |  |  |  | 6 | 8 | 15 | 9 | 6 | 2 | 7 | 16 | 5 |  |  |  | 4 | 64 | 8 |
|  |  |  |  |  |  |  |  |  |  |  | 8.1% | 10.8% | 20.3% | 12.2% | 8.1% | 2.7% | 9.5% | 21.6% | 6.8% |  |  |  |  |  |  |
| Enrofloxacin | 2 |  |  |  |  | 3 | 4 | 2 | 0 | 1 | 6 | 10 | 9 | 4 | 8 | 15 | 11 | 1 |  |  |  |  | 4 | 32 | 0.125 |
|  |  |  |  |  |  | 0.0% | 5.4% | 2.7% | 0.0% | 1.4% | 8.1% | 13.5% | 12.2% | 5.4% | 10.8% | 20.3% | 14.9% | 1.4% |  |  |  |  |  |  |  |
| Florfenicol | 16 |  |  |  |  |  |  |  |  |  |  |  | 1 | 4 | 21 | 26 | 11 | 5 | 0 | 4 | 2 |  | 16 | 64 | 16 |
|  |  |  |  |  |  |  |  |  |  |  |  |  | 1.4% | 5.4% | 28.4% | 35.1% | 14.9% | 6.8% | 0.0% | 5.4% | 2.7% |  |  |  |  |
| Imipenem | 4 |  |  |  |  |  |  |  | 7 | 12 | 20 | 5 | 13 | 12 | 5 |  |  |  |  |  |  |  | 0.5 | 4 | 0.5 |
|  |  |  |  |  |  |  |  |  | 9.5% | 2.7% | 27.0% | 6.8% | 17.6% | 16.2% | 6.8% |  |  |  |  |  |  |  |  |  |  |
| Colistin | 2 |  |  |  | 1 | 0 | 4 | 8 | 24 | 23 | 10 | 0 | 0 | 0 | 0 | 0 | 0 | 0 | 0 | 0 | 4 |  | 0.125 | 0.5 | 2 |
|  |  |  |  |  | 1.4% | 0.0% | 5.4% | 10.8% | 32.4% | 31.1% | 13.5% | 0.0% | 0.0% | 0.0% | 0.0% | 0.0% | 0.0% | 0.0% | 0.0% | 0.0% | 5.4% |  |  |  |  |
| Neomycin | 32 |  |  |  |  |  |  |  |  |  |  |  | 7 | 14 | 2 | 2 | 11 | 22 | 5 | 5 | 6 |  | 64 | 256 | 8 |
|  |  |  |  |  |  |  |  |  |  |  |  |  | 0.0% | 18.9% | 2.7% | 2.7% | 14.9% | 29.7% | 6.8% | 6.8% | 8.1% |  |  |  |  |
| ^4^Potentiated sulfonamide | 4 |  |  |  |  |  |  |  |  |  |  |  | 7 | 8 | 3 | 13 | 6 | 5 | 0 | 2 | 5 | 25 | 32 | 1024 | 0.5 |
|  |  |  |  |  |  |  |  |  |  |  |  |  | 9.5% | 10.8% | 4.1% | 17.6% | 8.1% | 6.8% | 0.0% | 2.7% | 6.8% | 33.8% |  |  |  |
| Spectinomycin | 128 |  |  |  |  |  |  |  |  |  |  |  |  |  |  | 7 | 18 | 23 | 7 | 13 | 1 | 5 | 64 | 256 | 64 |
|  |  |  |  |  |  |  |  |  |  |  |  |  |  |  |  | 9.5% | 24.3% | 31.1% | 9.5% | 17.6% | 1.4% | 6.8% |  |  |  |

^1^ Clinical Laboratory Standards Institute (CLSI); ^2^Epidemiological cut-off value (EUCAST); ^3^2:1 ratio; ^4^trimethoprim-sulfamethoxazole in ratio 1:19.
